# CRISPR/Cas9-mediated *PHOX2B* functional knock-out in IMR32 neuroblastoma cells impairs neuronal excitability through dysregulation of ion channels genes

**DOI:** 10.3389/fphys.2026.1844142

**Published:** 2026-06-24

**Authors:** Silvia Cardani, Martina Bertocchi, Erika Donà, Filippo Chiesa, Clara Cambria, Ana Lucia Cuadros Gamboa, Eleonora Piscitelli, Vladimir Rancic, Simon Gosgnach, Silvia Pagliardini, Flavia Antonucci, Diego Fornasari, Simona Di Lascio, Roberta Benfante

**Affiliations:** 1 Department of Medical Biotechnology and Translational Medicine (BIOMETRA), Università degli Studi di Milano, Milan, Italy; 2 Department of Physiology, Faculty of Medicine and Dentistry, University of Alberta, Edmonton, AB, Canada; 3 Women and Children’s Health Research Institute, University of Alberta, Edmonton AB, Canada; 4 National Research Council (CNR) - Institute of Neuroscience, Vedano al Lambro, MB, Italy; 5 NeuroMi - Milan Center for Neuroscience, University of Milano Bicocca, Milan, Italy; 6 National Research Council (CNR) - Institute of Biomedical Technologies, Segrate, MI, Italy; 7 Neuroscience and Mental Health Institute, University of Alberta, Edmonton, AB, Canada

**Keywords:** ChIP-seq, CRISPR/Cas9, ion-channels regulation, iPSC-derived autonomic neurons, neuroblastoma, neuronal excitability, PHOX2B, RNA-seq

## Abstract

**Introduction:**

PHOX2B is a master transcriptional regulator of autonomic nervous system development whose mutations cause neurocristopathies including neuroblastoma and congenital central hypoventilation syndrome (CCHS). However, the identification and functional relevance of PHOX2B target genes remain incompletely defined, partly due to the lack of robust cellular models.

**Methods:**

Through CRISPR/Cas9-engineering we established a PHOX2B functional knockout IMR32 neuroblastoma model, and combined transcriptomic, chromatin-binding, electrophysiological, and live-cell imaging analysis to investigate PHOX2B-dependent pathways. We then assessed the developmental relevance of the obtained results in iPSC−derived sympathetic neurons from CCHS patients carrying different PHOX2B mutations.

**Results:**

PHOX2B functional knockout (PHOX2B-KO) cells exhibited morphological and molecular features indicative of enhanced neuronal maturation. RNA-seq and ChIP-seq analyses highlighted an unexpected direct role for PHOX2B in regulating the expression of ion channels, including *KCNQ5, SCN3A* and *RYR2*, primarily through transcriptional repression. Functionally, PHOX2B KO cells displayed significant alterations in intrinsic electrical properties, including depolarized resting membrane potential, reduced action potential amplitude, attenuated Ca2^+^ responses and profoundly altered K^+^ efflux dynamics. Functional rescue experiments confirmed that PHOX2B re-expression restores both transcriptional and electrophysiological phenotypes, thereby establishing a causal relationship between PHOX2B expression and the maintenance of intrinsic excitability homeostasis. Extending these findings to development, sympathetic neurons differentiated from CCHS patient-derived iPSCs displayed dysregulated maturation, transcriptional network rewiring, and altered expression of the same ion channel genes, supporting their relevance to disease.

**Discussion:**

Together, our data establish the PHOX2B functional knockout neuroblastoma model as a valuable platform for functional genomics, enabling systematic analysis of PHOX2B targets and highlighting its critical role in coordinating transcriptional programs that shape neuronal excitability and maturation.

## 1 Introduction

The Autonomic Nervous System (ANS) develops through a hierarchical program of transcription factors that orchestrate the expression of specific cell surface molecules. These molecules mediate responses to extrinsic cues, including growth factors and components of the extracellular matrix, which in turn drive neurogenesis, gliogenesis, precursor cells migration, target innervation, synaptogenesis, and the acquisition of neurotransmitter identity (Ernsberger and Rohrer, 2018; Lefcort, 2020).

Among these regulators, PHOX2B plays a central role. This paired like homeobox transcription factor is highly conserved across mammals and is essential for the differentiation and maintenance of visceral sensory and motor neurons that regulate cardiovascular, respiratory, and digestive functions and constitute the sensory and motor components of visceral reflex circuits (Pattyn et al., 2000b; Pattyn et al., 1999, Pattyn et al., 1997; Di Lascio et al., 2018a). *PHOX2B* is also expressed in key chemosensory structures such as the carotid body and the area postrema (Dauger et al., 2003), and within the central nervous system (CNS) its expression encompasses all noradrenergic centers, cranial motor nuclei, and selected interneuron populations in the hindbrain and spinal cord (Pattyn et al., 1997; Pattyn et al., 2000b; Kang et al., 2007; Pattyn et al., 2000a). Peripherally, PHOX2B governs the development and differentiation of neural crest (NC)-derived structures, including autonomic ganglia and the adrenal medulla (Pattyn et al., 1999; Brunet and Pattyn, 2002).

Insights from *Phox2b*-null mouse models further highlight its critical role in ANS development. In these animals, all autonomic ganglia, the three cranial sensory ganglia involved in autonomic reflexes, and all neurons expressing noradrenergic markers fail to develop properly (Pattyn et al., 1999). Furthermore, PHOX2B controls the expression of key noradrenergic determinants, tyrosine hydroxylase (*TH*) and dopamine-β-hydroxylase (*DβH*), two key enzymes involved in noradrenaline biosynthesis (Tiveron et al., 2003; Ernsberger et al., 2000; Yang et al., 1998), and promotes neuronal maturation by coordinating cell cycle exit (Dubreuil et al., 2000).

In addition to its well-established developmental functions, PHOX2B remains expressed in several autonomic and brainstem nuclei, pointing to functions that extend beyond embryogenesis. In adult rats, *Phox2b* expression persists in hindbrain structures associated with orofacial movement, auditory processing, and ocular control, but not in rhythm-generating respiratory centers. In addition, it remains highly expressed in neurons of the retrotrapezoid nucleus (RTN) and dorsal vagal complex, both essential for the central and peripheral chemoreflex pathways (Kang et al., 2007; Di Lascio et al., 2021). This sustained expression in selected areas of the brain may be important for the maintenance of the noradrenergic phenotype (Cui et al., 2021; Fan et al., 2018; Zhu et al., 2005; Coppola et al., 2010) and of neurons regulating breathing homeostasis (Cardani et al., 2024; Cui et al., 2024) in adulthood.

PHOX2B has been shown to regulate its own expression (Cargnin et al., 2005) and a set of downstream targets primarily involved in lineage specification and neuronal survival and differentiation (reviewed in (Di Lascio et al., 2021).

Heterozygous mutations in PHOX2B - including both alanine tract expansions of a 20 alanine repeat in the C-terminal of PHOX2B protein (genotype 20/25 to 20/33; PARM) and non-polyalanine mutations (NPARM) - as well as dysregulated gene expression (Di Lascio et al., 2021; Di Lascio et al., 2013, Di Lascio et al., 2018b), are clinically linked to a group of congenital disorders known as neurocristopathies (Vega-Lopez et al., 2018). These disorders include Hirschsprung’s disease (HSCR; OMIM 142623) (Di Zanni et al., 2017; Fernández et al., 2013), neural crest-derived tumors such as neuroblastoma (NB) (Longo et al., 2008; Perri et al., 2005; Di Lascio et al., 2018b; Bourdeaut et al., 2005) and Congenital Central Hypoventilation Syndrome (CCHS, OMIM 209880) (Amiel et al., 2003; Weese-Mayer et al., 2017; Trang et al., 2020), all characterized by defects in neuronal development, maturation, or functional integration, including absence of enteric innervation in HSCR (Butler Tjaden and Trainor, 2013), arrested differentiation of sympatho-adrenal progenitor cells in NB (Schleiermacher et al., 2014) and failed formation of the RTN, a key chemoreceptor involved in respiratory control, in CCHS murine models (Dubreuil et al., 2008; Di Lascio et al., 2018a).

Transcriptional dysregulation mediated by loss-of-function (LOF; haploinsufficiency), dominant-negative, as well as gain-of-function (GOF) mechanisms through toxic mutant forms have been shown to contribute significantly to the pathogenesis of both CCHS and neuroblastoma (Cargnin et al., 2005; Di Lascio et al., 2018b; Di Zanni et al., 2012; Di Lascio et al., 2013; Boeva et al., 2017; Di Lascio et al., 2021). However, the broad transcriptional program governed by PHOX2B and its impact on neuronal function remains poorly defined, largely due to the lack of suitable cellular models that accurately represent PHOX2B-expressing lineages, and of systematic analyses of how this master-gene shapes intrinsic neuronal properties.

To overcome these limitations, we generated a *PHOX2B* functional knockout model in IMR32 neuroblastoma cells using CRISPR/Cas9-mediated engineering and combined RNA-seq, ChIP-seq, whole-cell electrophysiology, and live-cell Ca^2+^/K^+^ imaging to delineate PHOX2B-dependent pathways, whose developmental relevance were also assessed in iPSC−derived sympathetic neurons from CCHS patients carrying different PHOX2B mutations. The results from this multimodal approach revealed that PHOX2B directly regulates the expression of ion−channel genes that are required to maintain proper electrical properties and highlighted its critical role in coordinating transcriptional programs that shape neuronal excitability and maturation in both tumoral and developmental contexts.

## 2 Materials and methods

### 2.1 Cell cultures and generation of stable clones

The human neuroblastoma cell line IMR32 was grown in RPMI 1640 medium (Corning), supplemented with 10% fetal bovine serum (FBS, Corning), 100 units/mL penicillin, 100 mg/mL streptomycin, 2 mM L-glutamine (Corning). The IMR32 A6.4 *PHOX2B* KO stable clone was maintained under selection with 0.150 mg/mL Puromycin dihydrochloride (Sigma-Aldrich), with selective medium replaced every second day. The IMR32 A6.4 PHOX2B WT* stable clones were maintained under selection by adding Puromycin dihydrochloride (Sigma-Aldrich) and Geneticin (G-418 Sulphate, Gibco) at 0.150 mg/mL and 0.170 mg/mL final concentration, respectively. All cell lines grow in adhesion at 37 °C in a humified atmosphere containing 5% CO2.

*IMR32 A6.4 PHOX2B functional KO stable clone.* A *PHOX2B* functional knockout (KO) clone (A6.4) was generated using CRISPR/Cas9 (**Supplementary Figure 1**) (Komor et al., 2017). Two gRNAs targeting exon 1 of *PHOX2B* (PHOX2B-A: 5’ - GCAGGAACTGAAGTCAGCAT - 3’; PHOX2B-B 5’ -CATACAGGACTCGTAGGCAG - 3’) were designed and cloned into the pCLIP-All vector, containing a puromycin resistance cassette, by the Custom transEDIT CRISPR Design Service (TransOMIC Technologies Inc.).

gRNAs activity was validated with the GeneArt™ Genomic Cleavage Detection Kit (Life Technologies), according to the manufacturer’s instructions. IMR32 cells (1x10^6^) were transfected with 1 μg of *PHOX2B* gRNA plasmid using 3 μL of Lipofectamine 3000 (Life Technologies), selected with puromycin (0.250 mg/mL, 5 days), and extracted genomic DNA (Cell Lysis buffer/Protein Degrader, Invitrogen) was analyzed by PCR-based cleavage detection. A region of exon 1 of the *PHOX2B* gene was amplified using specific primers (forward: 5’ – GCTCGGTGCAGTATGAGTGTGGTA - 3’; reverse: 5’ - TCAGAAAAGTTGACCCAAACTC - 3’). PCR products were denatured and re-hybridized under step down annealing conditions to generate homo- and heteroduplex. The duplex mixture was digested with the Detection Enzyme for 1 hour at 37 °C and analyzed on a 2% agarose gel. For each sample, a no enzyme control was included. Cleavage efficiency was quantified using UVITEC Cambridge transilluminator and UVI-1D analysis software and expressed as the percentage of cleaved products relative to the total amplified DNA (parental plus cleaved fragments).

For stable clone generation, IMR32 cells were transfected with Sma I-linearized (New England BioLabs) gRNA vectors (5 μg DNA, 1: 3 DNA-Lipofectamine™ 3000 ratio). After puromycin selection, individual colonies were isolated, expanded, and screened by immunoblotting using N- and C-terminal PHOX2B antibodies (listed in **Supplementary Table 1**) (Cargnin et al., 2005). Gene editing was confirmed by Sanger sequencing. A scrambled-sequence gRNA (5’- GGAGCGCACCATCTTCTTCA -3’) was used as a control to exclude non-specific CRISPR/Cas9 activity, and the resulting clones were pooled for subsequent analysis.

The KO clone A6.4 was analyzed for morphological changes and for expression of glial, neuronal, and maturation markers by Western blotting and qPCR. Transcript stability was assessed following treatment with 5, 6 dichloro-beta-D-ribofuranosylbenzimidazole (75 μM; DRB, Sigma-Aldrich) for the indicated times. Statistical analyses were performed with GraphPad Prism 10 Software (GraphPad Software, Inc.) using non-linear regression analysis and unpaired two-tailed Student’s t-test; p<0.05 was considered statistically significant.

*IMR32 A6.4 PHOX2B WT stable clone.* Because IMR32 A6.4 *PHOX2B* functional KO stably express Cas9 and would cleave *PHOX2B WT* plasmid, the PAM sequence in the Myc-tagged *PHOX2B WT* construct was mutated (**Supplementary Figure 2A**, PHOX2B WT* pcDNA 3.1/Myc-His) to prevent Cas9 recognition (**Supplementary Figure 2B**). The generation of the Myc-tagged *PHOX2B WT* plasmid has been described previously (Bachetti et al., 2005a; Parodi et al., 2012; Di Lascio et al., 2013). Point mutations were introduced into the PAM sequence by recombinant PCR with specific primers, subcloned into pCR II-TOPO vector (Invitrogen), and sequence verified by sequencing. The oligonucleotides used to generate the mutations were #PAM MUT FW, 5’ – CTGGCTTCAG*C**A**T*ATGCTGACTTCAG - 3’ and #PAM MUT REV, 5’ – TCAGCAT*A**T**G*CTGAAGCCAGGCTC - 3’; the external primers were #T7 MOD FW 5’ – ACGACTCACTATAGGGAGACCCAAGC - 3’ and #*PHOX2B* ex2 REV 5’ – ACTCGCGCCTCTGTGAGGTCG - 3’ (PAM sequence is in italics and the changed nucleotide is underlined).

To generate a stable PHOX2B re-expressing line (IMR32 A6.4 PHOX2B WT), 1x10^6^ IMR32 A6.4 *PHOX2B* KO cells were plated in 100 mm dishes in antibiotic-free RPMI 1640 medium and transfected with 5 μg of a Myc-tagged, PAM mutated *PHOX2B* wild-type plasmid (PHOX2B WT* pcDNA 3.1/Myc-His) using Lipofectamine 3000 (DNA: Lipofectamine ratio 1:3). After 48 h, cells were diluted 1:10 and selected with 500 μg/mL geneticin. Resistant colonies were expanded and screened for PHOX2B expression by Western blot.

### 2.2 hPSC line and differentiation of iPSCs into sympathetic neurons

Human induced pluripotent stem cells (hiPSCs) were generated from fibroblast obtained from skin biopsies and characterized as described (Cuadros Gamboa et al., 2022, Cuadros Gamboa et al., 2026; Di Lascio et al., 2026), in compliance with ethical committee (number 7/2015) at the Meyer Children’s Hospital IRCCS, Florence, Italy. hiPSCs were cultured in Essential 8™ Flex medium (Gibco, Cat#A2858501) on Matrigel hESC-qualified coated dishes (Corning, Cat#CLS354277). Cells were passaged biweekly as clumps using an EDTA (Invitrogen, Cat#15575020) dissociation solution (0.5 mM EDTA/PBS) and maintained at 37 °C and 5% CO_2_. The cells were routinely tested for mycoplasma contamination, and their genomic integrity was periodically assessed through karyotyping.

iPSCs from the healthy donor line CTRL R1 (Di Lascio et al., 2026) and three CCHS patients UMILi027-A, UMILi028-A (Cuadros Gamboa et al., 2022) and UMILi034-A (Cuadros Gamboa et al., 2026), were differentiated into sympathetic neurons using a stepwise protocol designed to mimic *in vivo* sympathetic neuron development, as described (Di Lascio et al., 2026), with minor modifications. Briefly, using a modified dual SMAD inhibition strategy under adherent and chemically defined culture conditions, we single-cell plated iPSCs and induced trunk neural crest cells (NCCs) in a 10-days long process, by passaging them at day 3. On day 10, once full confluency is reached, cells were dissociated, re-plated and subjected to 4-days of Sympathoadrenal Progenitor (SAP) Induction, followed by 14-21-days of Sympathetic Neuron Maturation. Developmental progression was assessed by immunocytochemistry using PHOX2B, TH and TUBB3 antibodies, as described in (Di Lascio et al., 2026), and qPCR analyses. Primary and secondary antibodies are listed in **Supplementary Table 1**.

### 2.3 RNA extraction and gene expression analysis

Total RNA was extracted using *the RNeasy Mini kit* or *Micro kit* and QIAshredder (Qiagen). cDNA was synthesized from 0.25–0.5 μg RNA using the GoScript™ Reverse Transcriptase kit (Promega). qPCR was performed on a Quant Studio 5 system (Applied Biosystems, CA) using TaqMan^®^ assays (Life Technologies, Inc.) for *human Paired like homeobox 2B* (*PHOX2B*, ID #Hs00243679_m1), human *SRY-related HMG-box* (*SOX10*, ID #Hs00366918_m1), human *Homeobox C8* (*HOXC8*, ID # Hs00224073_m1), human *Homeobox C9* (*HOXC9*, ID # Hs00396786_m1), human *Islet-1* (*ISL1*, ID # Hs00158126_m1), human *Achaete-scute homolog 1* (*ASCL1*, ID # Hs00269932_m1), human *Peripherin* (*PRPH*, ID # Hs00196608_m1), human *Dopamine-β-hydroxylase* (*DBH*, ID Hs01089840_m1), human *choline acetyltransferase* (*ChAT*, ID #Hs00252848_m1), human *potassium voltage-gated channel subfamily Q member 5* (*KCNQ5*, ID # Hs01068536_m1), human *sodium voltage-gated channel, alpha subunit 3* (*SCN3A*, ID # Hs00366902_m1), human *ryanodine receptor-2* (*RYR2*, ID # Hs00181461_m1) and the endogenous control human *glyceraldehyde-3 phosphate dehydrogenase* (*GADPH*, ID #Hs99999905_m1). Each reaction was performed in triplicate. Relative expression was calculated with the 2^−ΔCT^ and the 2^−ΔΔCT^ methods.

For RNA-seq experiments, three independent RNA samples per condition were prepared and sent to IGA Technology Services s.r.l.(Udine, Italy) for analysis. Briefly, RNA samples quantified and quality tested by Tape Station RNA assay (Agilent Technologies, Santa Clara, CA) and RNAs with an RNA Integrity Number above 6 were processed for sequencing. Sequencing libraries were prepared from 200 ng of total RNA using the VAHTS Universal v10 RNA-Seq kit (Vazyme, Nanjing, PRC), according to manufacturer recommendation, and their quality checked with Qubit 3.0 Fluorometer Invitrogen, Carlsbad, CA) and Agilent Bioanalyzer DNA assay (Agilent technologies, Santa Clara, CA). 150 bp paired-end sequencing was performed on NovaSeq 6000 instrument (Illumina, San Diego, CA).

Reads were aligned to the human reference genome hg38-iGenomes genome using STAR (Dobin et al., 2013), with default parameters (https://github.com/alexdobin/STAR). Data were further processed in R using DEseq2 library (Love et al., 2014). Raw RNA-seq count matrices were assembled from per-sample read count files and filtered to remove genes with zero counts across all samples. A DESeq2 dataset object was constructed using sample condition as the design factor, and variance stabilizing transformation (VST) was applied blind to condition for exploratory analysis. Sample-level quality and relationships were assessed by PCA on the VST-transformed data, and PC loadings were inspected to identify the genes driving the main axes of variation. Expression of curated noradrenergic and neuronal differentiation marker genes was visualized by hierarchical clustering heatmaps. Differential expression was then computed with DESeq2 across all pairwise condition combinations — Control vs. KO, Control vs. KO_rescue_1, Control vs. KO_rescue_2, KO vs. KO_rescue_1, and KO vs. KO_rescue_2 — using an adjusted p-value threshold of 0.05. To identify transcriptionally rescued genes, we extracted genes that were differentially expressed between Control and KO and showed a consistent reversal in both re-expression conditions compared to KO and were no longer significantly different from the Control in either rescue condition. Overlaps across comparisons were visualized using Euler diagrams (eulerr, https://cran.r-project.org/package=eulerr). For data mining and visualization, we used the tidyverse (Wickham et al., 2019) and ggplot2 (Wickham, 2016) R packages.

### 2.4 Chromatin immunoprecipitation and massive parallel sequencing analyses

ChIP was performed as previously described using chicken anti-hPHOX2B antibodies (Davids Biotechnologie, Ragensburg, Germany (Cargnin et al., 2005)). Immunoprecipitated DNA was quantified by qPCR using *Power* SYBR-Green PCR Master Mix (Applied Biosystems) on a QuantStudio 5 system (Applied Biosystems, CA) with primers listed in **Supplementary Table 2**. *PHOX2B* primers (Cargnin et al., 2005) amplifying promoter and 3’-UTR regions were included as positive and negative control, respectively. Library preparation and sequencing were performed by Genomnia s.r.l. on the SOLiD^®^ 5500 W platform (Applied Biosystems). Reads were mapped to the human genome (UCSC Genome Browser (GRCh37/h19) using the Lifetech Lifescope 2.5.2 software, following “a priori” error correction with the SAET procedure (Nanni et al., 2013). Alignment files (pairwise Input and Experiment) were analyzed for peak calling using MACS version 1.4.2 (Zhang et al., 2008). Exploratory analysis of sequencing quality, MACS peak metrics and peak annotation was performed with the Integromics SeqSolve bioinformatic software suite. Correlation of MACS peaks (Experiment versus Input) with annotated NCBI RefSeq gene structures was carried out using in-house Genomnia Perl scripts and with the ChIPpeakAnno Bioconductor package, version 2.15.1 (Zhu et al., 2010).

Comparison with publicly available PHOX2B ChIP-seq datasets was performed using Integrated Genome Viewer (IGV) (http://www.broadinstitute.org/igv/). PHOX2B-ChIP-seq profiles from BE2C cells (GEO ID: GSM2486153) and Kelly cells (GEO ID: GSM2915910) were obtained from (Durbin et al., 2018); PHOX2B-ChIP-seq data from CLBGA cells (GEO ID: GSM2664369) were obtained from (Boeva et al., 2017); PHOX2B-ChIP-seq data from IMR32 transfected with siPHOX2B (GEO ID: GSM5576949) or siCTRL (GEO ID: GSM5576946) were obtained from (Xu et al., 2023).

Motif discovery within ChIP-seq peaks of PHOX2B was performed using the Position Analysis module of the RSAT-peak motifs suite (oligonucleotide size: 6 nt) (Thomas-Chollier et al., 2012).

Gene ontology enrichment analysis was carried out using PANTHER version 19.0 (https://pantherdb.org/webservices/go/overrep.jsp) (Thomas et al., 2022) applying the GO-slim molecular function, cellular component and biological process function ontologies. Statistical significance was assessed using Fisher’s exact test, and p-values were adjusted using either FDR or Bonferroni multiple testing correction.

### 2.5 Protein preparation and Western blot analyses

Total protein was extracted from sub-confluent cells using a freeze-thaw lysis method and analyzed by Western blotting (Benfante et al., 2008). Primary and secondary antibodies are listed in **Supplementary Table 1**. Detection was performed with LiteAblot Extend Long Lasting Chemiluminescent Substrate (Euroclone) and imaged using ChemiDoc™ Imaging System (Bio-Rad). Protein ladders from Thermo Fisher Scientific (PageRuler™ Broad Range Unstained Protein Ladder) and Euroclone (Prestained Protein SHARPMASS TMVII) were included as molecular weight standards. Densitometric analysis was performed with Image Lab 6.1 (Bio-Rad).

### 2.6 *In vitro* electrophysiology and live-cell imaging

Whole-cell current clamp recordings were performed using an Axopatch 200B amplifier and pClamp-10 software (Axon Instruments). Recordings were performed in Krebs’-Ringer’s-HEPES (KRH) solution (NaCl 125 mM, KCl 5 mM, MgSO_4_ 1.2 mM, KH_2_PO_4_ 1.2 mM, CaCl_2–_2 mM, glucose 6 mM, HEPES-NaOH pH 7.4–25 mM). Recording pipettes (3–5 MΩ) were filled with potassium-gluconate-based intracellular solution (KGluc 130 mM, KCl 10 mM, EGTA 1 mM, HEPES 10 mM, MgCl_2–_2 mM, MgATP 4 mM, GTP 0.3 mM). Cells were subjected to 25 current injections (-120 pA to +360 pA in 20 pA increments) to assess passive and active electrical properties. Data were analyzed using Clapfit-pClamp 10 software (Molecular Devices).

For live cell K^+^ and Ca^2+^ imaging, 2.5x10^5^ cells (IMR32, IMR32 A6.4 *PHOX2B* KO, and IMR32 A6.4 PHOX2B WT* cl 1 and cl 2) were plated on poly-L-lysine-coated coverslips (Millipore-Sigma) and transfected with 250 ng each of K-GECO1 (Ca^2+^ sensor (Shen et al., 2018)) and GINKO1 (K^+^ sensor (Shen et al., 2019)) using 1:3 DNA: Lipofectamine™ 3000 ratio. After 18–24 h, imaging was performed in 20 mM HEPES buffered Hank’s balanced salt (HBSS) solution (HHBSS). After baseline acquisition, cells were stimulated with Nicotine (1 mM, 1 min; Millipore-Sigma). Images were acquired every 2 s for 13 min using a XLUMPlan Olympus 20x immersion objective (1.0 numerical aperture). Fluorescence signals were analyzed using MetaMorph (Molecular Devices) and Image J. Regions of interest (ROIs) corresponding to individual cells were manually defined, and fluorescence intensity traces were extracted over time. Signals were normalized and expressed as ΔF/F_0_, where F_0_ represents the baseline fluorescence measured prior to stimulation. For each experiment, approximately 40 cells per condition were analyzed. Single-cell data within each experiment were averaged to yield one value per condition. Experiments were performed in six independent biological replicates performed on separate days.

For Ca^2+^ responses, the following parameters were quantified: peak amplitude (maximum ΔF/F_0_), time-to-peak (interval from stimulation onset to peak), and area under the curve (AUC), calculated relative to baseline over the post-stimulation period.

For K^+^ responses, given the sustained decrease in signal without attainment of a stable plateau within the recording window, the following parameters were quantified: maximum K^+^ response (defined as the absolute minimum ΔF/F_0_ following stimulation), mean ΔF/F_0_ during the final time window (720–780 s), and AUC relative to baseline over the post-stimulation period.

### 2.7 Statistics

Details of the statistical analyses are provided in figures legends. Data are expressed as mean ± SD or SE, as indicated in figure legends. Statistical analyses were performed using GraphPad Prism 10.6.0 (GraphPad Software, Inc.) p<0.05 was considered significant.

## 3 Results

### 3.1 Generation and characterization of a PHOX2B knockout model in IMR32 cells

To investigate PHOX2B-dependent pathways, we used the human neuroblastoma cell line IMR32, which endogenously expresses PHOX2B, and generated a CRISPR/Cas9-mediated knockout line. The gRNAs targeting *PHOX2B* were designed to avoid the homeodomain (HD) region, which shows high homology with other homeobox proteins (Galliot et al., 1999). We selected two 20-bp candidate gRNAs, named *PHOX2B-A* and *PHOX2B-B*, positioned immediately at the 5’ of the trinucleotide proto-spacer adjacent motif (PAM) sequence on the minus strand of the *PHOX2B* gene (**Supplementary Figure 1A**), and tested their efficiency and specificity at the target site using a cleavage detection assay (**Supplementary Figure 1B**). These gRNAs were used to generate stable KO clones. The clones obtained were screened for PHOX2B protein expression by Western blot analysis using two different antibodies directed against the N- and C- terminal regions of the protein, to avoid false negative results (**Supplementary Figure 1C**). Most clones showed specific bands recognized by both antibodies, with molecular weights either higher or lower than control (**Supplementary Figure 1C**), suggesting the presence of insertions or deletions at the cleavage site leading to truncated or elongated PHOX2B protein products. Four clones showed a complete absence of PHOX2B protein compared with naïve IMR32 (data not shown and **Supplementary Figure 1C**). To characterize the gene editing events in these putatively knockout clones, we sequenced the PCR-amplified exon 1 region. Among all sequenced clones, only clone A6.4, hereafter referred to as *PHOX2B* KO or KO, carried a homozygous mutation (**Supplementary Figure 1D**), resulting in loss of protein expression (**Supplementary Figure 1E**). Despite the absence of PHOX2B protein, *PHOX2B* mRNA level was slightly increased in the mutant clone (**Supplementary Figure 1F**, white bar vs black bar). This phenomenon is frequently reported in CRISPR-edited systems, likely due to increased transcript stability and escaped complete degradation by nonsense-mediated decay (NMD) (**Supplementary Figure 1G**), supporting a functional knockout (Lindeboom et al., 2019; Kim et al., 2024).

**Figure 1:**
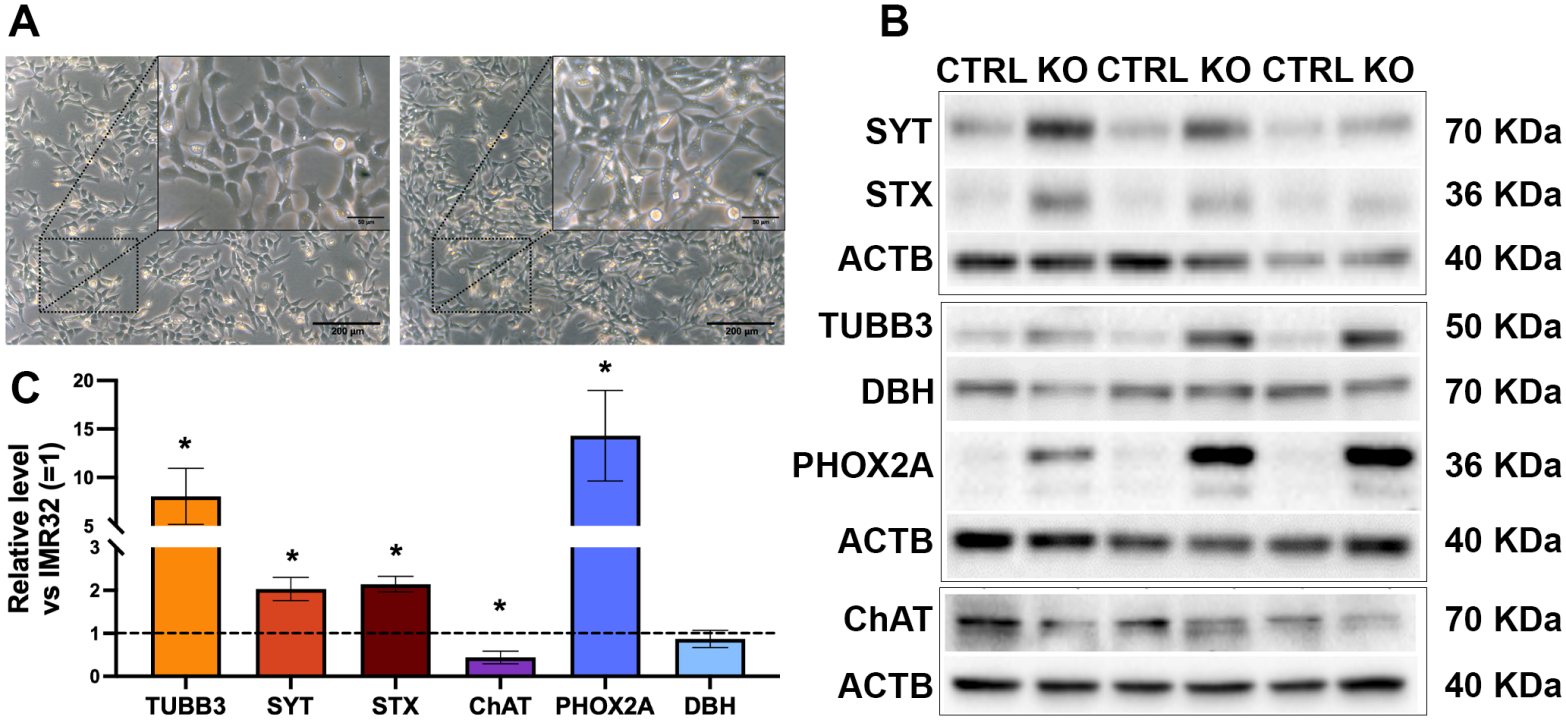
Molecular and cellular characterization of IMR32 A6.4 *PHOX2B* KO cells. **(A)** Phase−contrast images comparing native IMR32 cells (left) and the IMR32 A6.4 *PHOX2B* KO clone (right). High−magnification insets (top) correspond to the boxed areas in the lower−power fields. **(B)** Representative Western blots showing the expression of synaptotagmin (SYT), Sintaxin 1A (STX), ß3-tubulin (TUBB3), dopamine- ß-hydroxylase (DBH), PHOX2A and choline acetyltransferase (ChAT), in total cell lysates from IMR32 (CTRL) and clone A6.4. (KO). 20 μL of cellular extract were separated by means of 10% SDS-PAGE and transferred to a nitrocellulose membrane. β-actin (ACTB) was used as a normalizer. **(C)** Relative protein quantification in IMR32 A6.4 *PHOX2B* KO cells vs IMR32 (=1, dotted line). Bars show mean value (± SEM, error bars) of three independent samples. *p < 0.05, significant differences in protein expression between *PHOX2B* KO cells and IMR32 naïve cells (one-tailed Mann-Whitney test).

Given the role of PHOX2B in the specification of cell type identity, we next investigated whether its absence induced changes in cellular morphology or in the expression of glial and neuronal markers. Qualitative inspection of phase-contrast images (**Figure 1A**) suggests that *PHOX2B* KO cells may exhibit morphological differences compared with IMR32 control, including more extended processes and elongated cell bodies, potentially indicative of a more differentiated neuronal phenotype; however, these observations are descriptive, as no quantitative analysis was performed. Consistent with previous reports in neuroblastoma models (Yang et al., 2020), qPCR analysis showed no detectable transcript of the neural crest-derived glial cell marker *SOX10* in either naïve IMR32 cells or KO cells (data not shown), indicating that IMR32 cells, which are committed to a neuronal lineage (SOX10^-^/PHOX2B^+^), do not revert to a glial-like phenotype upon PHOX2B loss (Nagashimada et al., 2012). Thus, removing PHOX2B in IMR32 cells is not sufficient to drive a switch toward a SOX10^+^/PHOX2B^-^ glial fate, despite their differentiation block along the sympathetic neuronal trajectory.

Expression of the neuronal marker β3-tubulin (TUBB3) was increased in clone A6.4 compared with IMR32 cells, thus confirming that PHOX2B loss promotes progression of differentiation along the neuronal lineage, leading to a more mature phenotype (**Figures 1B, C**). This interpretation is supported by increased levels of synaptotagmin (SYT) and syntaxin (STX) proteins, two vesicular markers involved in docking and priming of synaptic vesicles, as well as increased expression of PHOX2A, the paralog of PHOX2B involved in noradrenergic specification. As expected for neuroblastoma cells (Bourdeaut et al., 2009), both cholinergic (ChAT) and catecholaminergic (DBH) markers were detected. Loss of PHOX2B resulted in reduced ChAT protein levels while leaving DBH expression unchanged, indicative of a more mature noradrenergic phenotype (**Figures 1B, C**).

**Figure 2:**
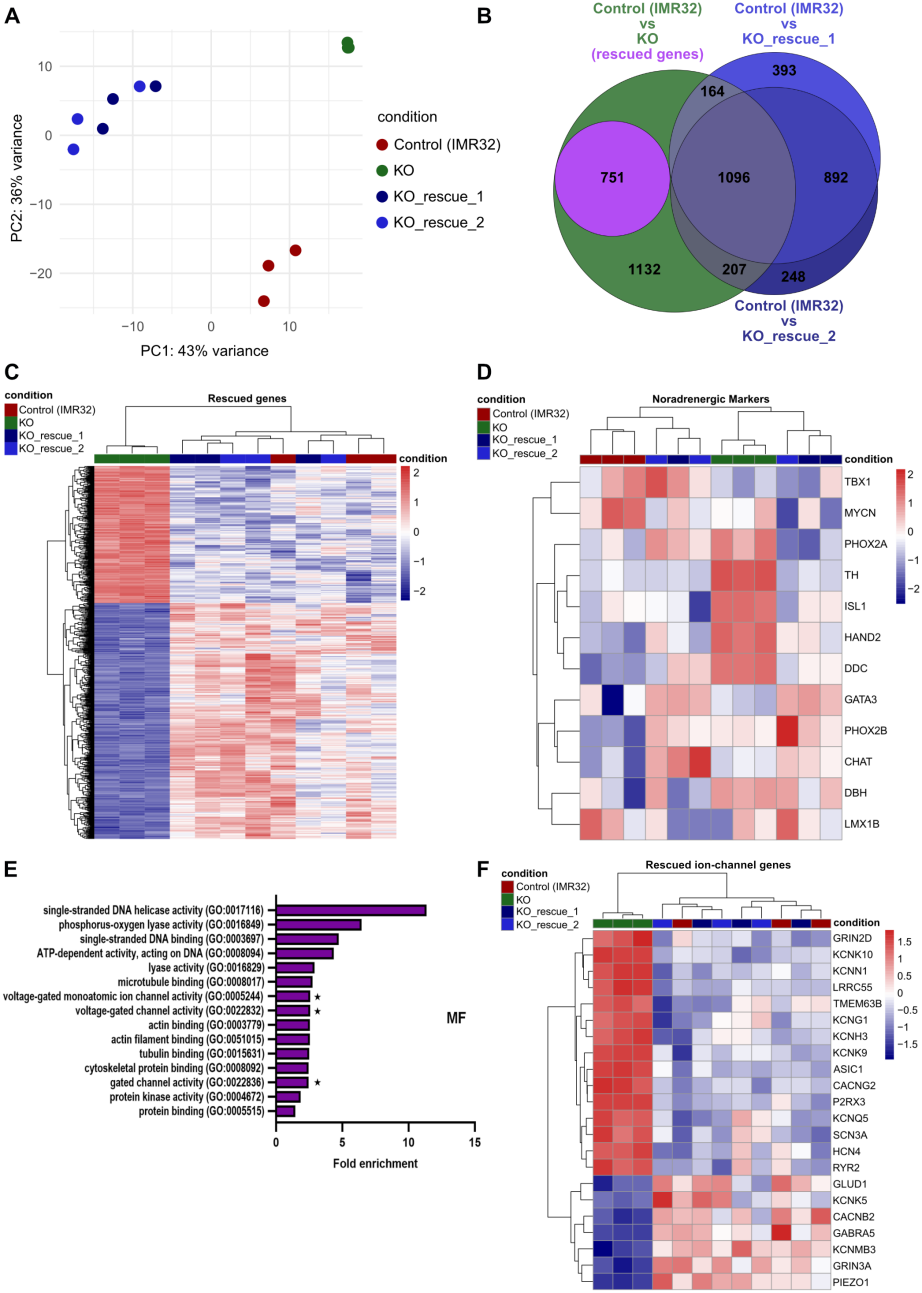
RNA-seq identifies ion channels as candidate PHOX2B-regulated genes. **(A)** Principal component analysis (PCA) showing separation of samples by condition: IMR32 control (red), *PHOX2B* KO (green), and two independent PHOX2B WT*-rescue clones (KO_rescue 1, dark blue; KO_rescue 2, light blue). **(B)** Venn diagram illustrating the overlap between genes differentially expressed (DE) in control (IMR32 cells) vs. *PHOX2B* KO (green) and those DE in CTRL vs. each rescue clone (blue). The magenta region highlights genes altered in the KO but restored to control levels in both rescue lines, representing candidates for direct PHOX2B regulation. **(C)** Heatmap of expression patterns for the rescued gene set identified in panel **(B)**. **(D)** Heatmap showing expression profiles of canonical noradrenergic markers and the cholinergic marker *ChAT* across all conditions. **(E)** Top 15 gene Ontology (molecular function, MF) terms enriched among the 751 rescued genes, with asterisks marking categories related to ion channels. **(F)** Heatmap displaying the expression of rescued ion-channel genes belonging to the starred categories in panel **(E)**.

These results were corroborated by RNA-seq experiments, aimed at investigating the transcriptional consequences of PHOX2B loss in the IMR32 neuroblastoma cell line (**Figure 2**). Because we sought to determine which RNA-seq-derived candidate genes were directly regulated by PHOX2B, we generated two independent rescue lines stably re-expressing wild-type PHOX2B in the IMR32 A6.4 background (IMR32 A6.4 PHOX2B WT*, **Supplementary Figures 2A, B**; hereafter referred to as KO_rescue). Two independent clones (cl 1 and cl 2, hereafter named KO_rescue_1 and KO_rescue_2), expressing comparable levels of PHOX2B protein, were selected for subsequent analyses (**Supplementary Figure 2C**). Principal Component Analysis (PCA) confirmed that the two rescue clones were transcriptionally homogeneous (**Figure 2A**). However, PHOX2B re-expression did not revert the transcriptional profile to that of naïve IMR32 cells, suggesting that *PHOX2B* KO cells may have undergone irreversible differentiation programs (**Figure 2A**). Moreover, a pool of scrambled gRNA transfected clones did not show transcriptional changes comparable to those observed in *PHOX2B* KO cells (data not shown), indicating that these effects were not due to nonspecific CRISPR/Cas9 activity or clonal selection.

We reasoned that genes directly regulated by PHOX2B should be differentially expressed between IMR32 and KO cells, but not between IMR32 and the rescue condition. Differential expression analysis identified 1883 genes differentially expressed between IMR32 and PHOX2B KO (**Figure 2B**), 751 of which were rescued by PHOX2B re-expression (**Figures 2B, C**). Rescued genes included *HAND2* and *ISL1* (**Figure 2D**), lineage-specific transcription factors that together with PHOX2B form a specific Core Regulatory Circuitry (CRC) critical for establishing and maintaining the noradrenergic phenotype and are known direct PHOX2B targets (Durbin et al., 2018; Boeva et al., 2017; Zhang et al., 2018; Hendershot et al., 2008; Schmidt et al., 2009). While the up-regulation of these genes in the absence of PHOX2B may initially appear unexpected (Durbin et al., 2018; Boeva et al., 2017), this is compatible with disruption or re-programming of the auto-regulatory loops involving PHOX2B and its downstream targets (Zimmerman et al., 2021), which could result in misexpression due to compensatory transcriptional feedback from other genes including paralogous genes, such as PHOX2A (**Figures 1B, C**) and GATA 2 (Shendy et al., 2022), and induction of cell differentiation. Notably, re-expression of PHOX2B in the knockout clone partially restored *PHOX2A* expression levels toward those observed in control cells (**Supplementary Figure 3**), consistent with the known functional redundancy and cross-regulatory interactions between PHOX2 family members during autonomic neuron development (Di Lascio et al., 2016b; Flora et al., 2001; Coppola et al., 2010). In addition, sequence alignment analysis revealed multiple mismatches between the PHOX2B-targeting gRNAs and the PHOX2A locus (data not shown), making a CRISPR/Cas9 off-target effect highly unlikely.

Gene ontology (GO) enrichment analysis of the 751 rescued genes highlighted ion channel-related categories among the most significantly represented terms (**Figure 2E**). Indeed, a large number of ion channel genes, including voltage-gated potassium, sodium and calcium channels, were deregulated in PHOX2B KO cells, and restored toward control levels in both rescue clones (**Figure 2F**), indicating their PHOX2B-dependent transcriptional regulation. These findings suggest that cellular ion balance might be altered in the absence of PHOX2B.

### 3.2 PHOX2B modulates the electrical properties of neuroblastoma IMR32 cell line

Based on the observed increase in neuronal maturation markers in *PHOX2B* KO cells and on the altered expression of several ion channel genes, we wondered whether the electrical properties of IMR32 cells were affected by PHOX2B loss.

**Figure 3:**
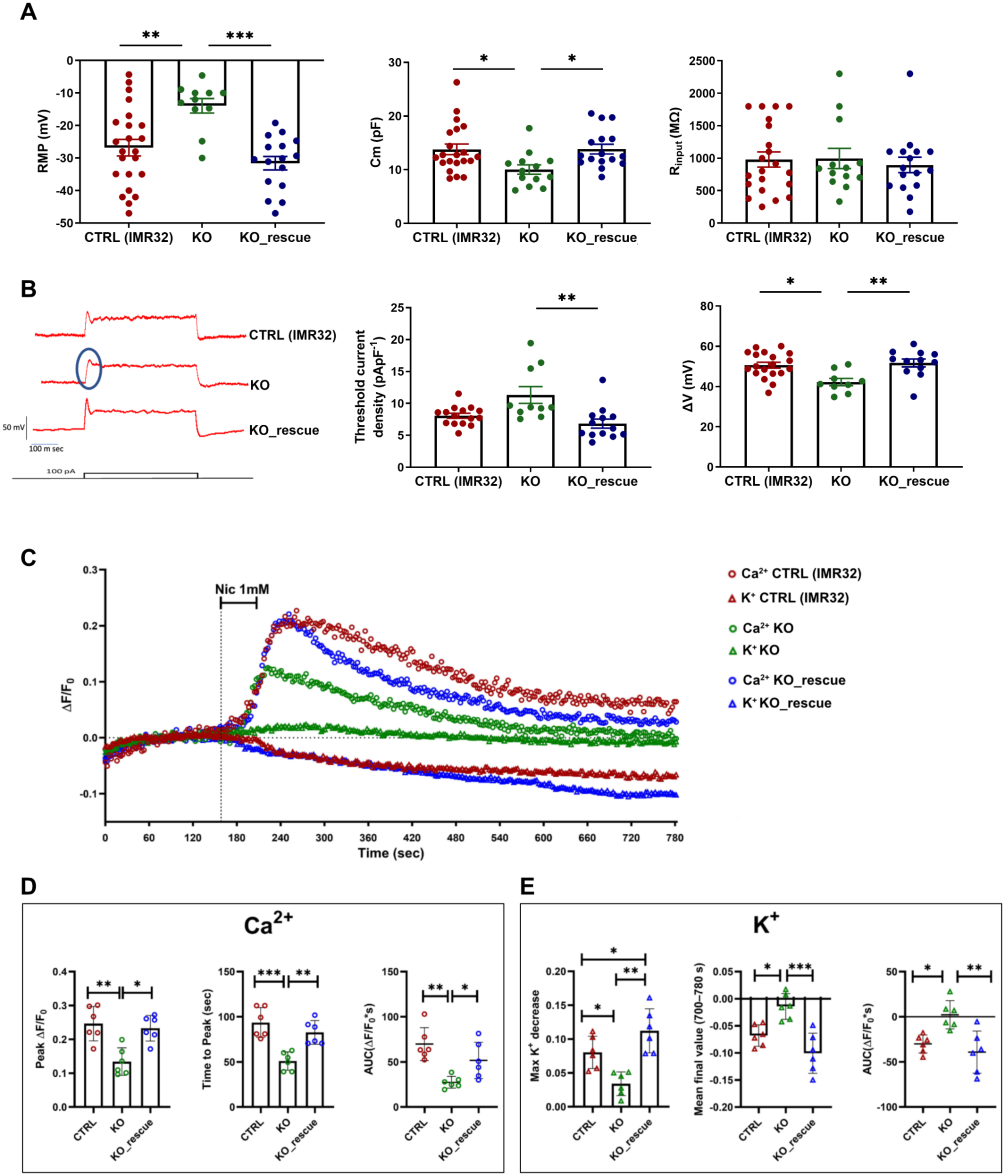
Characterization of electrical properties in IMR32 A6.4 *PHOX2B* KO cells. **(A)** Analysis of passive membrane properties of IMR32 (red dots), KO (green dots) and KO_rescue (blue dots) cells through current clamp recordings. In comparison with IMR32 and KO_rescue, KO cells display an increased RMP value (Ordinary one-way ANOVA followed by Tukey’s multiple comparisons test: *** p<0.001; n° of cells recorded: IMR32 = 23, KO = 11, KO_rescue = 16) and a reduced Cm value (Kruskal-Wallis test followed by Dunn’s multiple comparisons test: ** p<0.01; n° of cells recorded: IMR32 = 21, KO = 13, KO_rescue = 16). No differences in terms of R_input_ were found (Kruskal-Wallis test followed by Dunn’s multiple comparisons test: ns; n° of cells recorded: IMR32 = 21, KO = 13, KO_rescue = 16). Data are expressed as mean ± SEM. **(B)** Representative traces and analysis of active membrane properties of IMR32, KO and KO_rescue cells through current clamp recordings. KO cells display a reduced excitability in contrast with IMR32 and KO_rescue cells: the threshold current density of KO is higher compared to KO_rescue cells (Kruskal-Wallis test followed by Dunn’s multiple comparisons test: **p<0.01; n° of cells recorded: IMR32 = 15, KO = 10, KO_rescue = 13) and the action potential amplitude (ΔV) is reduced (Kruskal-Wallis test followed by Dunn’s multiple comparisons test: ** p<0.01; n° of cells recorded: IMR32 = 19, KO = 9, KO_rescue = 12). Data are expressed as mean ± SEM. **(C)** Representative mean normalized fluorescence traces (ΔF/F0) of time course of Ca^2+^(K-GECO1, circles) and K^+^ (GINKO1, triangles) responses to nicotine stimulation (1 mM, 60 s) in naïve IMR32 cells (red), KO cells (green), and KO_rescue (blue). Traces represent the mean signal across cells within each experiment, averaged over six independent biological replicates. **(D)** Quantification of Ca^2+^ responses, including peak amplitude (ΔF/F0), time to peak, and area under the curve (AUC), showing attenuated Ca^2+^ signaling in KO cells and restoration upon PHOX2B re-expression. **(E)** Quantification of K^+^ responses, including maximal decrease in ΔF/F0 (ΔK^+^ max), mean ΔF/F0 during the final time window, and AUC, demonstrating impaired K^+^ efflux in KO cells and enhanced responses in KO_rescue cells. Data are presented as mean ± SD of independent biological replicates. Statistical significance was assessed by one-way ANOVA followed by Tukey’s *post-hoc* test. *p<0.05, **p<0.01, ***p<0.001.

To this end, we performed current clamp recordings to characterize passive and active membrane properties in naïve IMR32 cells, *PHOX2B* KO cells and cells re-expressing the WT form of PHOX2B (clones KO_rescue 1 and KO_rescue 2). We first compared the passive membrane properties (i.e., resting membrane potential (RMP) Vm at I = 0, capacitance Cm and input resistance R_input_) obtained at resting. As shown in **Figure 3A**, IMR32 cells, as immature neurons, displayed a more depolarized RMP than mature neurons. Moreover, PHOX2B KO cells exhibited a less negative RMP and reduced Cm, despite having comparable input resistance. This suggests altered ionic homeostasis, potentially due to disrupted K^+^ channel function or changes in ion transporter expression, within an immature cellular context suggested by the reduced Cm. Since K^+^ conductance typically dominates at rest, a less negative RMP may reflect reduced K^+^ efflux or increased inward current from other ions. No differences were detected between control IMR32 cells and KO_rescue clones, thus confirming that PHOX2B plays a role in maintaining the cellular electrical passive properties.

A more depolarized RMP should result in a reduced amplitude of the action potential (AP), due to the generation of a smaller electrochemical gradient (Nadasdy et al., 1998). We therefore evaluated intrinsic excitability properties of cells, injecting hyperpolarizing-to-depolarizing current step-wise starting from the resting potential in current-clamp whole-cell mode (20 pA/step, 25 sweeps ranging from -120 pA to +360 pA). We found that the minimal current density (current/Cm) necessary to induce the generation of an action potential in KO cells showed a trend toward higher values compared to IMR32 cells, although this difference did not reach statistical significance (**Figure 3B**). In contrast, PHOX2B re-expressing cells displayed normal values of minimal current density. Measurement of deltaV (action potential amplitude – ΔV) measured upon depolarizing currents was significantly lower in the PHOX2B KO cells compared with naïve IMR32 (CTRL) and KO_rescue cells. Overall, these data indicate reduced excitability in cells lacking PHOX2B, consistent with the depolarized membrane potential and decreased action potential amplitude.

To corroborate these findings, we performed live cell Ca^2+^ and K^+^ imaging using genetically encoded and fluorescent-based calcium (K-GECO1) and potassium (GINKO1) ions indicators (Shen et al., 2018; Shen et al., 2019). Upon depolarizing stimulation with 1 mM nicotine, naïve IMR32 cells displayed robust Ca^2+^ influx accompanied by a marked decrease in intracellular K^+^ levels (**Figure 3C**). In contrast KO cells exhibited significantly attenuated responses with slower kinetics, characterized by reduced Ca^2+^entry, a lower peak ΔF/F_0_ and a decreased area under the curve (AUC), despite reaching peak levels more rapidly than control cells (**Figure 3D**).

K^+^ imaging revealed an even more pronounced defect: whereas control cells showed a sustained decrease in intracellular K^+^ following stimulation, KO cells displayed only a minimal reduction from baseline, indicating impaired K^+^ efflux (**Figure 3C**). Quantitative analysis confirmed a significantly smaller maximal ΔF/F_0_ decrease and reduced AUC in KO cells (**Figure 3E**).

Re-expression of PHOX2B restored K^+^ responses and resulted in a significantly greater maximal decrease compared with naïve IMR32 cells, indicating enhanced K^+^ efflux upon rescue (**Figures 3C, E**). PHOX2B re-expression also restored Ca^2+^ responses in KO_rescue cells to levels comparable to controls (**Figures 3C, D**).

Together, these data demonstrate that PHOX2B deficiency disrupts stimulus-evoked ion dynamics, leading to diminished Ca^2+^ signaling and defective K^+^ efflux, consistent with the altered membrane properties and reduced excitability observed in electrophysiological recordings. Moreover, restoration of PHOX2B rescues both Ca^2+^ and K^+^ transients to control levels, confirming that the impaired ionic responses observed in KO cells are specifically attributable to PHOX2B loss.

### 3.3 Ion channels are newly identified PHOX2B target genes

Since our results demonstrated that changes in PHOX2B protein levels modulates the electrical properties of IMR32 neuroblastoma cells, and RNA-seq analysis highlighted that the expression of ion channel genes is affected by PHOX2B depletion, we next investigated which of these genes are direct PHOX2B targets.

**Figure 4:**
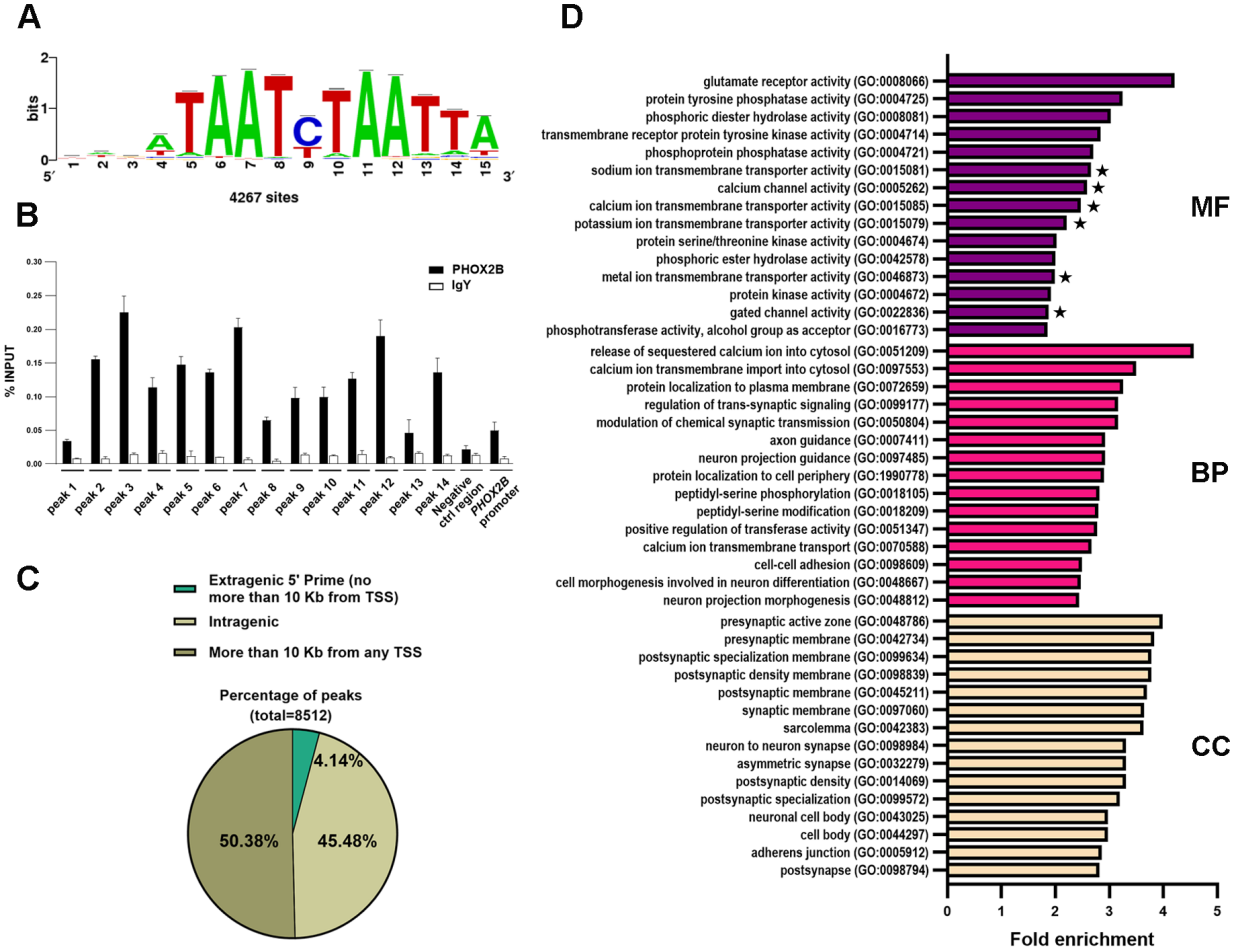
ChIP-seq analysis in IMR32 cell line identifies PHOX2B target genes. **(A)** PHOX2B recognition motif predicted using data from ChIP-seq experiments. **(B)** ChIP-qPCR validation at 14 representative peaks. IgY (PI, white bars) and PHOX2B (black bars) antibodies. A positive and negative control was included, corresponding to the amplification of *PHOX2B* promoter and 3’-UTR regions, respectively. Data are expressed as % of input. **(C)** Genomic distribution of PHOX2B-binding sites relative to Transcription Start Sites (TSS) according to human RefSeq genes (GRCh37/hg19, February 2009). **(D)** Gene ontology (GO) enrichment analysis of the PHOX2B associated peaks (MF - Molecular Function, BP - Biological Process, CC - Cellular Component) using Panther version 19.0. Asterisks indicate categories related to ion channels.

To identify PHOX2B-bound regulatory regions potentially influencing neuronal excitability, we performed PHOX2B ChIP-seq analysis in IMR32 cells (**Figure 4**). This analysis revealed 8512 PHOX2B ChIP-seq peaks. Motif enrichment identified an inverted arrangement of two consensus motifs for homeodomain proteins (ATTA sequence) in more than 50% of the peaks (**Figure 4A**), consistent with the already reported PHOX2B binding motifs (Boeva et al., 2017), and with cooperative DNA-binding behavior described for other paired-type homeodomain transcription factors (Cain et al., 2025). Since a relatively high background was observed with the PHOX2B antibody, we validated ChIP-seq peaks by selecting a set of 14 representative binding sites of various peak location (near TSS, intronic or intragenic) and testing by independent ChIP followed by qPCR analysis. All selected sites were validated (**Figure 4B**), confirming that the obtained PHOX2B binding profile is reliable. Peaks distribution analysis showed that 4.14% of PHOX2B-binding sites were located within 10 Kb upstream of transcription start sites, 45.48% were intragenic and the remaining binding sites (50.38%) mapped to distal intergenic regions (more than 10 Kb from any TSS) (**Figure 4C**), indicating that PHOX2B binds both promoter-proximal and enhancer-like regulatory region. This distribution is consistent with previous reports that PHOX2B, together with other core regulatory circuitry (CRC) transcription factors such as HAND2, ISL1, GATA3, TBX2, and MYCN, preferentially binds enhancer regions (Durbin et al., 2018).

Gene Ontology (GO) enrichment analysis of PHOX2B-associated genes has revealed strong enrichment for ion-channel expression, including sodium, potassium, and calcium ion transport, as well as key categories related to synaptic transmission, axon guidance, and pre- and post-synaptic structures (**Figure 4D**).

**Figure 5:**
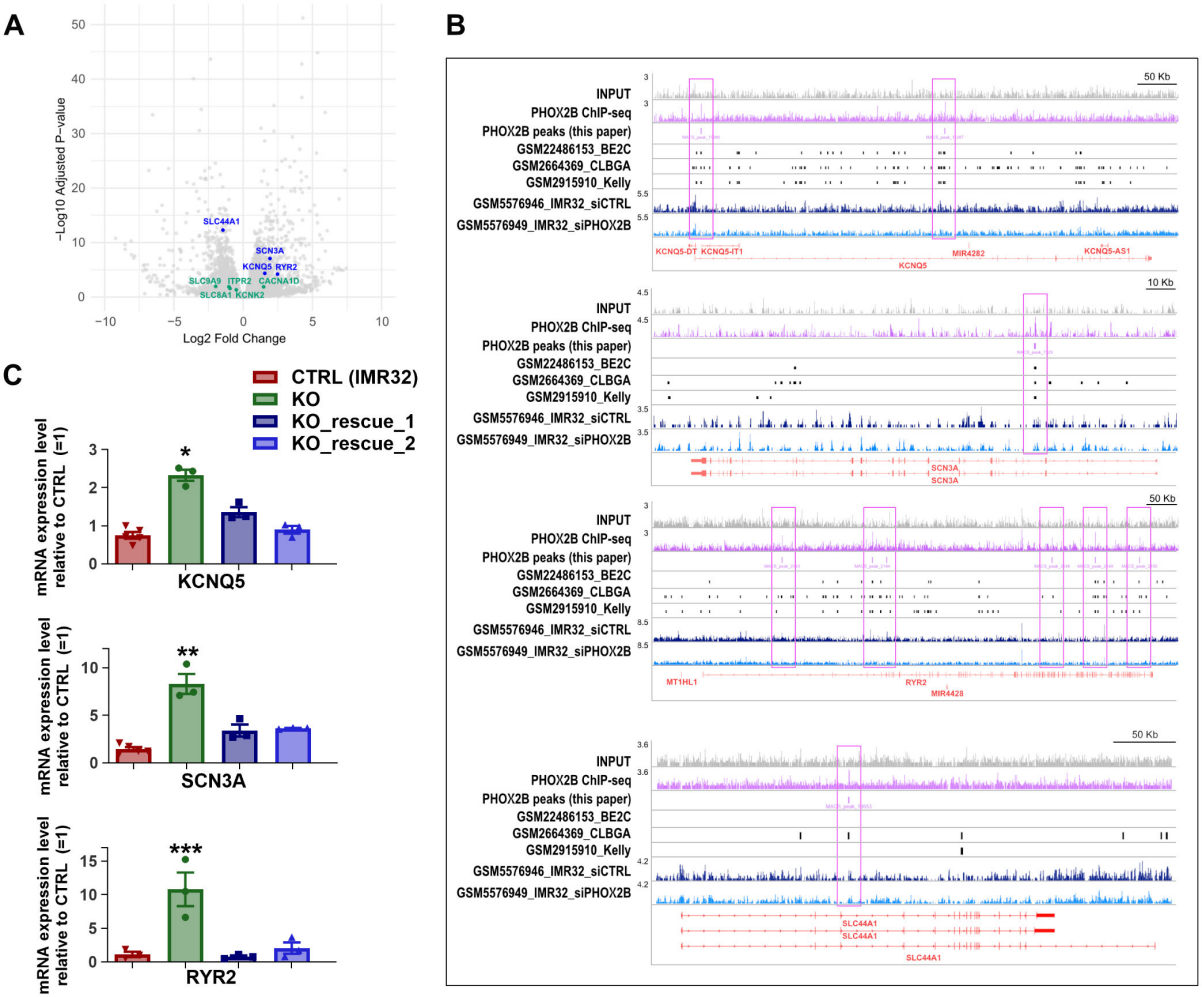
ChiP-seq and RNA-seq intersection analysis identify *KCNQ5, SCN3A, RYR2* and *SCL44A1* as PHOX2B target genes. **(A)** Volcano plot showing the results of differential expression analysis between KO cells and IMR32 (genes upregulated have positive log2 changes). Common genes between RNA-seq and ChIP-seq analysis belonging to ion channel categories are highlighted. *Green:* genes belonging to the 1883 group (green + magenta in **Figure 2B**). *Blue:* genes rescued by the re-expression of PHOX2B WT* protein. **(B)** Genome browser views of PHOX2B binding at *KCNQ5*, *SCN3A*, *RYR2*, and *SLC44A1* genes, compared with published ChIP-seq datasets in other neuroblastoma cell lines (Boeva et al., 2017; Durbin et al., 2018). Peaks that overlap between our dataset and published datasets are boxed in magenta, indicating concordant PHOX2B binding sites. **(C)** Transcriptional role of PHOX2B on ion channels encoding-genes. qPCR analysis of endogenous *KCNQ5, SCN3A, RYR2* mRNA in naïve IMR32 (red bar), KO (green bar) and KO_rescue 1 and 2 (blue bars). Data are normalized to that of the *GAPDH* gene. The bars are the mean values ± SEM (error bars) of three independent experiments and are expressed as fold relative to IMR32 native cells (=1). The 2^−ΔΔCT^ method was used to calculate the results. *p< 0.05, **p< 0.01, ***p<0.001, statistically significant differences in mRNA levels respect to IMR32 cells (one-way ANOVA, Tukey’s test).

Intersection of the 119 genes belonging to the ion and gated channel GO_Molecular Function (MF) categories from ChIP-seq data with the 751 genes obtained from RNA-seq data analysis (**Figures 2B, C**) identified *KCNQ5 (K_v_7.5)*, *SCN3A (Na_v_1.3), RYR2* and *SLC44A1* (*CLT-1*) as direct PHOX2B targets. Specifically, *KCNQ5, SCN3A and RYR2* were negatively modulated by PHOX2B (i.e. expression increased in PHOX2B KO), whereas *SLC44A1* was positively regulated (**Figure 5A**, blue dots). Moreover, PHOX2B ChiP-seq peak locations in these genes aligned with previously published datasets obtained in other neuroblastoma cell lines (Boeva et al., 2017; Durbin et al., 2018), further supporting the validity of our findings (**Figure 5B**). Beyond these four genes, extending the same analysis to other ion channel genes altered in PHOX2B-KO cells, that include *CACNA1D, ITPR2, KCNK2, SLC8A1*, and *SLC9A9* (**Figure 5A**, green dots), further supports the ChIP-seq dataset and a PHOX2B-dependent regulatory pattern (**Supplementary Figure 4**).

Consistently, qPCR shows that PHOX2B depletion significantly increases *KCNQ5, SCN3A* and *RYR2* mRNAs levels (**Figure 5C** green bars) while PHOX2B re-expression reduces them toward control in both rescue clones (**Figure 5C**, blue bars), thus confirming a PHOX2B-dependent transcriptional effect on these ion channel genes. Altogether, these data identify multiple ion channel classes as novel PHOX2B targets and mechanistically link PHOX2B-dependent transcription to intrinsic cell excitability.

### 3.4 PHOX2B mutations disrupt the coordinated progression of sympathetic lineage commitment and ion channels expression

**Figure 6:**
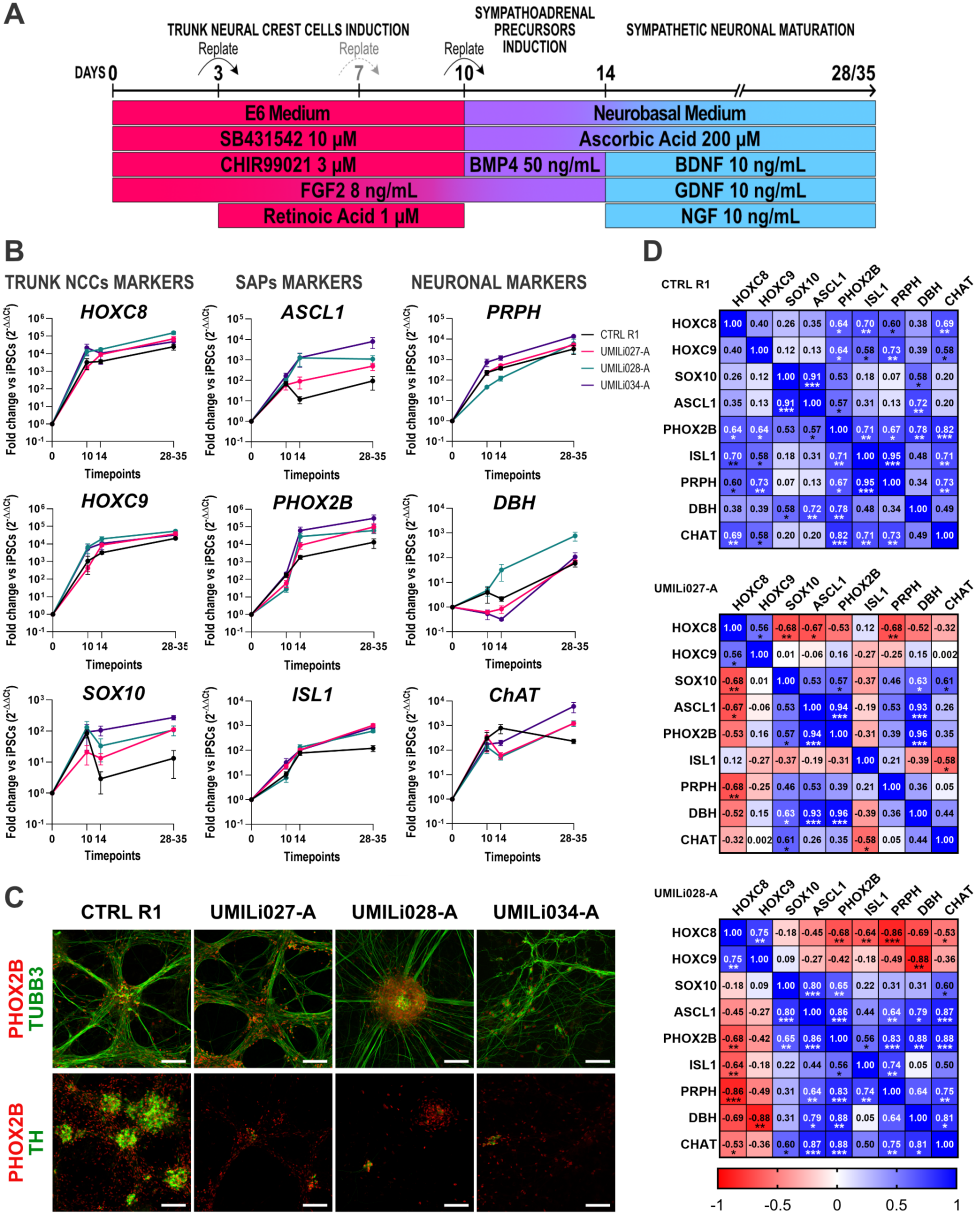
Differentiation of iPSC-derived sympathetic neurons. **(A)** Schematic overview of the sympathetic neuron differentiation protocol. Key stages, treatments, and durations are indicated. **(B)** qPCR analysis of the indicated markers was performed at the following time points: 0, 10, 14, 28, and 35 days, to track gene expression changes during *in vitro* differentiation (CTRL R1 N= D0, D10: 8, D14, D28-D35: 14; UMILi027-A N= D0, D10: 6, D14: 11, D28- D35: 14; UMILi028-A N= D0, D10: 7, D14: 12, D28- D35: 16; UMILi034-A N= D0, D10: 3, D14, D28- D35: 4). Results are presented as mean ± SEM and are expressed as fold change vs iPSCs (D0). **(C)** Representative immunostaining images of 35-days iPSC-derived sympathetic neurons stained for PHOX2B, TUBB3, and TH. Scale bars = 100 μm. **(D)** Spearman correlation matrices of autonomic neuron marker expression in control and CCHS patient-derived iPSC neurons highlights disrupted transcriptional architecture in mutant lines. Controls exhibited structured positive correlations consistent with hierarchical developmental progression, whereas CCHS lines showed partial uncoupling, including negative correlations between HOXC genes and sympathetic markers. Heatmaps show pairwise Spearman correlation coefficients computed from 2^-ΔCt^ values obtained by qPCR analysis of Day 35 sympathetic neurons. CTRL R1 and UMILi027-A N = 14, UMILi028-A N = 16. *p<0.05, **p<0.01 and ***p<0.001.

Given the transcriptional and network alterations identified in the PHOX2B KO model, and recognizing the central role of PHOX2B in sympathoadrenal development and its involvement in CCHS, along with the functional importance of ion channels in neuronal maturation, we next investigated whether the expression of *KCNQ5, SCN3A* and *RYR2* is modulated during differentiation and affected by PHOX2B mutations. To this end, we generated sympathoblasts (Sympathoadrenal progenitors-SAPs) and sympathetic neurons from hiPSCs, using a previously established protocol ( (Di Lascio et al., 2026) and **Figure 6A**). In particular, we used a control line (Control #R1 (Di Lascio et al., 2026)) and three hiPSC lines derived from CCHS-patients: (UMILi027-A, UMILi028-A (Cuadros Gamboa et al., 2022): genotypes 20/25 with late (LO-CHS) vs congenital onset; UMILi034-A (Cuadros Gamboa et al., 2026): genotype 20/26).

Although sympathetic neurons were previously derived from CCHS patients hiPSC lines (Amer-Sarsour et al., 2024), the transcriptional profiling of those neurons had not been characterized. Both control and mutant lines successfully activated early neural crest regulators *SOX10*, *HOXC8* and *HOXC9* by day 10, indicating preserved lineage competence (**Figure 6B**). However, temporal profiling revealed an impaired consolidation of sympathetic identity in mutant cells rather than complete lineage failure: *SOX10* and *ASCL1* remained elevated, despite higher *PHOX2B* levels, whereas maturation markers, including *PRPH* and *DBH* showed attenuated activation (**Figure 6B**). The persistence of *ChAT* expression in mutant lines suggested partial maintenance of the cholinergic program. Immunofluorescence further showed diminished TH staining and morphological alterations (**Figure 6C**). Notably, images with DAPI counterstaining (**Supplementary Figure 5**) confirmed the nuclear localization of PHOX2B, including in mutant cell lines. In the CTRL R1 line PHOX2B^+^ cells formed ganglion-like arrangements, as previously reported (Wu et al., 2022), and TUBB3 was organized in well-defined bundle fascicles, consistent with advanced neuronal maturation. In UMILi027-A, cells appeared less clustered, and TUBB3 exhibited a honeycomb-like mesh rather than compact bundles. In UMILi028-A, the clusters were larger, and TUBB3 appeared less organized. In UMILi034-A, both PHOX2B^+^ cell organization and TUBB3 architecture were disrupted, indicating a more pronounced alteration in neuronal network assembly.

This interpretation is corroborated by correlation gene network analysis performed on sympathetic neurons (**Figure 6D**). In CTRL R1 correlations are largely positive and structured, consistent with a hierarchical, temporally coordinated transcriptional program. In mutant lines, negative correlations between *HOXC* genes and *PHOX2B, SOX10, ISL1, PRPH* and *DBH*, indicate that axial identity genes are inversely coupled to the sympathetic cascade, suggesting a rewiring of gene-gene relationship. Despite robust *PHOX2B* induction during differentiation, its integration within the developmental network was weakened, with reduced coupling to downstream markers such as *ISL1*, leading to a less symmetric transcriptional cascade. This indicates that PHOX2B is present, but its integration into the developmental network is weakened indicating a network-level dysfunction, not simply expression-level reduction. Overall, the altered coupling between early neural crest, autonomic specification, and terminal differentiation markers suggests a disruption in the coordinated progression of sympathetic lineage commitment.

**Figure 7:**
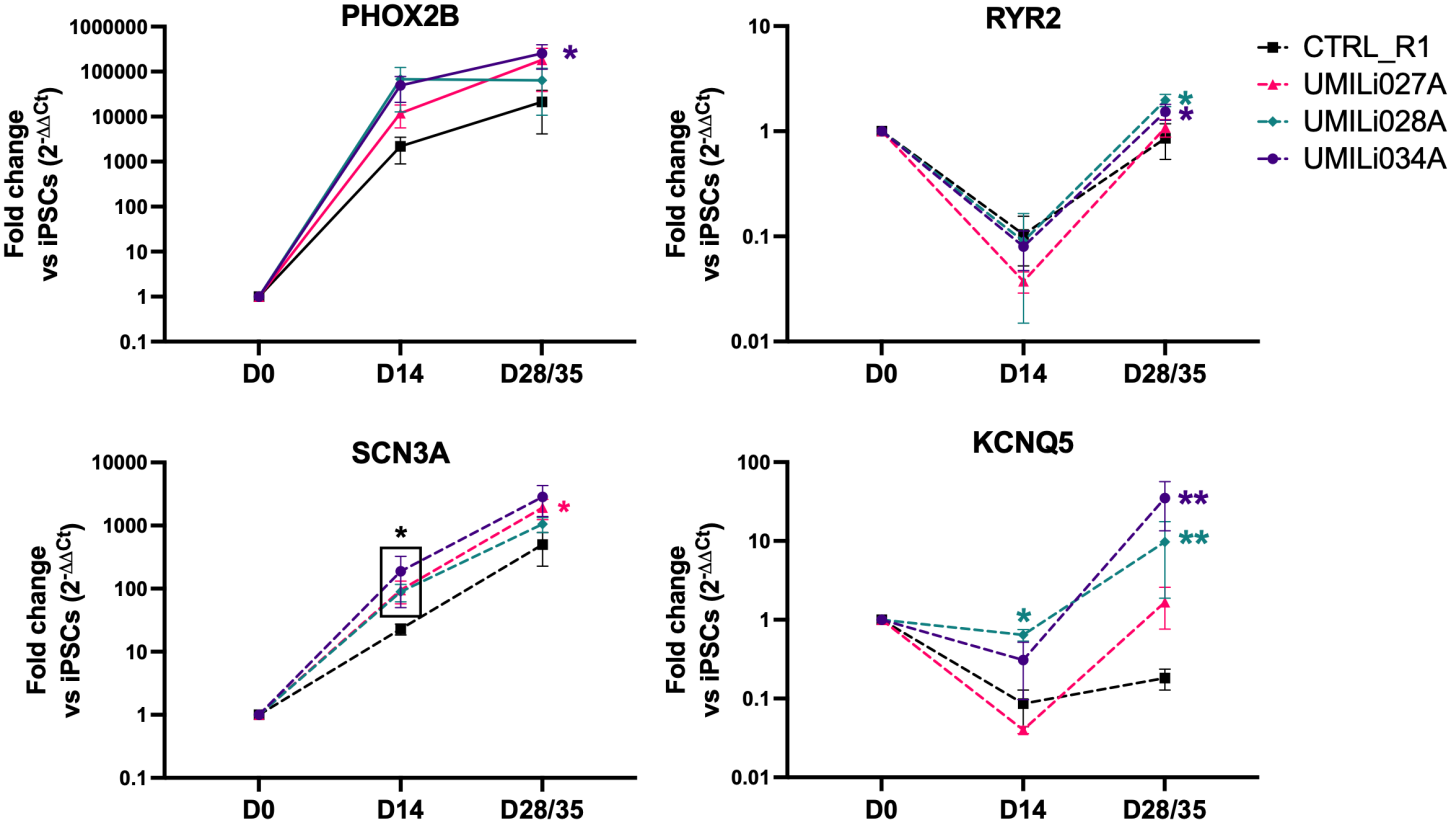
PHOX2B-dependent regulation of *RYR2, SCN3A* and *KCNQ5* during differentiation of control and mutant iPS-derived sympathetic neurons. A: qPCR analyses showing temporal expression of *PHOX2B* (solid line), *RYR2, SCN3A*, and *KCNQ5* (dashed lines) at 14 and 28–35 days during *in vitro* sympathetic differentiation of a control line R1 (black), and three CCHS-patients derived iPS cell lines: UMILi027-A (magenta), UMILi028-A (green) and UMILi034-A (purple). Data are mean of independent differentiation experiments (CTRL R1: N = 6; UMILi027-A and UMILi034-A: N = 5, UMILi028-A: N = 4), represented as fold change versus iPS cells (day 0) ± SEM. *p<0.05, **p<0.01 versus CTRL R1 cell line (one-tailed Mann-Whitney test).

Given the transcriptional and network defects observed, we next analyzed the expression of *KCNQ5, SCN3A* and *RYR2* in this cellular model. In the CTRL R1 line, *RYR2* and *KCNQ5* decreased at day 14, which corresponds to the sympathoadrenal progenitor (SAP) stage, followed by a subsequent increase at later stages (day 28/35), when cells reach terminal sympathetic neuron differentiation. Conversely, *SCN3A* showed a progressive increase from D0 to D35, paralleling the temporal induction of *PHOX2B* expression during differentiation. In the CCHS lines, we observed a similar temporal pattern (**Figure 7**). However, differences in expression levels were observed across individual patient lines, particularly at later differentiation stages (**Figure 7**).

At day 14, *RYR2* showed a comparable reduction in all CCHS lines, slightly greater, though not statistically significant, in UMILi027-Aand despite higher *PHOX2B* levels compared to control. A statistically significant increase in *SCN3A* was observed in all CCHS lines, potentially associated with elevated PHOX2B levels (although not significant).

*KCNQ5* level showed a statistically significant smaller decrease in congenital PARM 20/25 line compared with CTRL R1 and UMILi027-A (LO-CHS). The 20/26 UMILi034-A line, although exhibiting behavior similar to UMILi028-A, did not reach statistical significance due to high variability among biological replicates, highlighting the need to increase the number of replicates. At later stages, these differences become more sustained in particular for *KCNQ5*, which may reflect aberrant regulation, compensatory mechanisms, or altered protein function rather than simple dosage effects. Overall, our data provide the first evidence that, in a physiological context, mutant proteins carrying shorter PARM expansions exhibit transcriptional defects that were not previously described in overexpression models (Di Lascio et al., 2013; Bachetti et al., 2005b).

## 4 Discussion

In this study, we identify a previously underappreciated role for PHOX2B in the transcriptional control of ion channel genes and in the regulation of neuronal excitability in neuroblastoma cells. Although PHOX2B is well recognized as a master regulator of autonomic neuron development and noradrenergic differentiation (Pattyn et al., 1999; Ernsberger and Rohrer, 2018), its role in shaping electrophysiological properties during neuronal maturation and beyond has remained largely unexplored. Here, we show that PHOX2B restrains the expression of several ion channels genes, including *KCNQ5 (Kv7.5), SCN3A* (*Nav1.3*) and *RYR2*, contributing to maintain proper electrical activity and stimulus responsiveness. Modulation of these genes is also evident during sympathetic differentiation and is altered by *PHOX2B* mutations. In addition, regulation of the choline transporter *SLC44A1* suggests that the PHOX2B transcriptional program also extends to metabolic pathways supporting neuronal function and growth.

### 4.1 Loss of PHOX2B does not affect IMR32 noradrenergic identity

*PHOX2B* loss in IMR32 cells promotes neuronal maturation, evidenced by upregulation of TUBB3, synaptotagmin and sintaxin, suggestive of enhanced cytoskeletal organization and synaptic vesicle cycling capacity. While IMR32 cells co-express cholinergic and catecholaminergic markers, PHOX2B KO reduces ChAT without affecting DBH, indicating a shift toward a more defined noradrenergic identity (Chaudhari et al., 2017). This is consistent with studies showing that *PHOX2B* silencing or deletion does not abolish noradrenergic identity in neuroblastoma cells (Boeva et al., 2017; Thirant et al., 2021), despite previous reports linking PHOX2B expression loss to a mesenchymal-like phenotype (Boeva et al., 2017; Durbin et al., 2018). In contrast to our data, maintenance of noradrenergic identity has been associated with reduced expression of noradrenergic Core Regulatory Circuit (CRC) genes (*ISL1*, *GATA3* and *HAND2*) known to enforce lineage state (Boeva et al., 2017; Thirant et al., 2021). This discrepancy may reflect cell line differences or compensatory PHOX2A up-regulation, which can sustain noradrenergic specification and maintain this transcriptional network in the absence of PHOX2B (Coppola et al., 2010; Thirant et al., 2021). Similar transcriptional plasticity occurs during all-trans retinoic acid (ATRA)-induced differentiation, where early down-regulation of MYCN, PHOX2B and GATA3 is followed by CRC rewiring via epigenetic modulation of enhancer activity, sustaining HAND2 and ISL1 expression without altering lineage identity (Di Lascio et al., 2016b; Zimmerman et al., 2021). Consistently, *PHOX2B* KO cells showed reduced *MYCN*, normally downregulated during sympathetic neurons maturation (Knoepfler et al., 2002), and not restored by PHOX2B re-expression.

Overall, PHOX2B depletion promotes progression along a neuronal differentiation trajectory rather than dedifferentiation, suggesting a dual role as a lineage-determinant and temporal brake on terminal differentiation, preserving progenitor-like properties when required (Coppola et al., 2010). During development, this may ensure proper neuronal migration, target innervation, and circuit integration (Dubreuil et al., 2000; Dubreuil et al., 2002; Reiff et al., 2010), while in adulthood, PHOX2B likely contributes to maintaining terminal neuronal features (Cardani et al., 2024; Souza et al., 2023), consistent with age-related decline of catecholaminergic markers in rat locus coeruleus and adrenal glands following reduced *PHOX2A/PHOX2B* expression (Zhu et al., 2005).

### 4.2 PHOX2B controls intrinsic electrical homeostasis through repression of ion channel programs

Loss of PHOX2B markedly altered intrinsic electrical properties of IMR32 cells. KO cells exhibited a depolarized resting membrane potential, reduced membrane capacitance, preserved input resistance, a trend toward increased spike threshold, and significantly reduced action potential amplitude (ΔV), consistent with altered ion conductance. These changes were fully rescued by PHOX2B re-expression, demonstrating that they result specifically from PHOX2B loss.

Among the observed changes, impaired K^+^ handling appears to represent the primary driver of this phenotype. K^+^ imaging revealed a reduced efflux in KO cells following nicotine stimulation, consistent with a decrease in outward K^+^ conductance. Given the dominant role of K^+^ permeability in setting the resting membrane potential, this defect likely underlines the depolarized Vm observed in KO cells. Altered Ca^2+^ dynamics may further contribute to this phenotype. Reduced Ca^2+^ transients could limit the activation of Ca^2+^-dependent K^+^ channels, thereby further decreasing outward currents and reinforcing membrane depolarization.

RNA-seq and ChIP-seq analyses revealed a mechanistic basis for these effects. PHOX2B directly binds regulatory regions of multiple ion channel genes, including Na_v_ *(SCN3A)*, K_v_7 (*KCNQ5)*, and intracellular Ca^2+^ release machinery (*RYR2)* genes. These findings are consistent with previous studies employing PHOX2B ChIP-seq experiments in neuroblastoma cells (Boeva et al., 2017; Durbin et al., 2018), which, however, had not investigated functional consequences of these interactions. PHOX2B deletion increased *SCN3A, KCNQ5* and *RYR2* expression.

Transcriptional upregulation of *SCN3A* may enhance persistent Na^+^ conductance at subthreshold potentials, potentially influencing membrane depolarization and sodium channel availability through steady-state inactivation, thereby lowering action potential amplitude (Cummins et al., 2001). However, in the absence of direct electrophysiological measurements of Na^+^ currents, this contribution remains speculative. Similarly, increased *KCNQ5* may enhance M-current, acting as a stabilizing brake on excitability and increasing firing thresholds (Brown and Passmore, 2009; Hernandez et al., 2008). As Kv7 channels primarily regulate subthreshold excitability rather than rapid K^+^ efflux during stimulation, this does not contradict the impaired K^+^ dynamics observed by imaging.

Concurrently, *RYR2* up-regulation may alter Ca^2+^ dynamics, reducing recruitment of outwards currents during stimulation, contributing to the observed slower, diminished Ca^2+^ and K^+^ responses (Berridge, 2012). Together, these findings identify PHOX2B as a key regulator of cellular excitability, primarily through modulation of K^+^ homeostasis, with additional contributions from Ca^2+^ and Na^+^ signaling pathways. However, as gene expression changes do not necessarily translate into functional currents, direct electrophysiological validation will be required.

The convergence between PHOX2B and ion-channel regulation may also have implications for both neurocristopathies and neuroblastoma. Ion channels regulate proliferation, migration, differentiation, and morphology in both normal and cancer cells. Proliferating cells are typically depolarized, with membrane potential oscillations during the cell cycle (Levin, 2014). Increased K^+^ currents promote cell proliferation, while increased Na^+^ channel expression correlates with metastatic potential (Fan and Huang, 2022; Rosendo-Pineda et al., 2020). Calcium signaling via channels such as RYR2 has also been associated with improved survival in neuroblastoma patients (Lange et al., 2019). Persistent or dysregulated PHOX2B activity may therefore contribute not only to lineage identity but also to the aberrant electrical phenotype of tumor cells.

### 4.3 PHOX2B mutations determine a developmental delay during sympathetic neurons differentiation

Ion channels are critical for autonomic nervous system development, where early spontaneous electrical activity shapes intrinsic excitability and synaptic connectivity (Bates, 2015; Moody and Bosma, 2005). Premature excitability or Ca^2+^ oscillation can disrupt transcriptional programs governing migration, synaptogenesis, survival signaling and cell fate (McKinney and Kulesa, 2011; Pai et al., 2015; Zhong et al., 2019). In this context, PHOX2B-mediated repression of *SCN3A, KCNQ5* and *RYR2* may maintain controlled excitability set-point compatible with proper developmental progression (Smith and Walsh, 2020), while linking transcription to Ca^2+^ dependent signaling (Lobos et al., 2021; Bertan et al., 2020; Chiantia et al., 2023).

Human iPSC-derived sympathetic differentiation recapitulates a hierarchical transcriptional program linking neural crest identity (SOX10^+^) to autonomic specification (PHOX2B^+^, ASCL1^+^), and terminal maturation markers (ISL1^+^, PRPH^+^, DBH^+^). In control cells, these markers formed a coherent transcriptional network consistent with coordinated lineage progression (Zhang et al., 2018; Ernsberger and Rohrer, 2018). In contrast, CCHS-derived lines (UMILi027-A, UMILi028-A) displayed partial uncoupling of this developmental program, indicating rewiring of regulatory interactions despite robust *PHOX2B* induction. In particular, *HOXC8* and *HOXC9* correlated strongly with each other but negatively with sympathetic markers, suggesting destabilized interactions between axial patterning and autonomic specification. These observations support the idea that CCHS mutations impair PHOX2B functional output, disrupting the hierarchical organization of the transcriptional cascade (Di Lascio et al., 2013; Nagashimada et al., 2012). Despite preserved terminal markers *DBH* and *PRPH* expression, altered correlations and persistent *ChAT* expression suggest delayed maturation timing rather than complete lineage failure, consistent with evidence that partial ISL1 deficiency in sympathetic ganglia increases cholinergic gene expression (Zhang et al., 2018).

Ion channel expression dynamics further support this model. In control cells, *RYR2* and *KCNQ5* were transiently repressed around *PHOX2B* induction, and recovered at later stages, suggesting temporal gating of electrophysiological maturation during lineage specification (McKinney and Kulesa, 2011). *SCN3A* expression increased during differentiation, consistent with its high fetal CNS expression and role in early excitability and subsequently decline postnatally, as other voltage-gated sodium channels become more dominant (Liao et al., 2023), although a negative modulation of its expression cannot be excluded by time-course analysis.

In mutant lines, increased *SCN3A*, *KCNQ5* and *RYR2* expression indicates loss of proper transcriptional regulation and premature excitability. For *SCN3A* and *RYR2* this may partially be attributable to increased *PHOX2B* levels. In contrast, the altered behavior of *KCNQ5* may reflect aberrant regulation, compensatory mechanisms, or altered protein function rather than simple dosage effects. These findings indicate that, while the PHOX2B KO model reveals ion channel genes directly repressed by PHOX2B, mutant PHOX2B proteins in CCHS cells exert allele-specific and context-dependent effects that are not equivalent to complete loss of function. The lines used in this study derive from patients with isolated CCHS; polyalanine expansion mutations generate a stable protein (Di Lascio et al., 2013; Di Lascio et al., 2016a; Pirone et al., 2019) that may retain partial activity, interfere with the wild-type allele, or acquire novel regulatory properties, thereby uncoupling transcript levels from functional output. Future studies in lines bearing NPARM variants linked to syndromic form of the disease (CCHS + neuroblastoma; (Di Lascio et al., 2018b)), will be needed to expand our findings on PHOX2B-mediated deregulation of these genes.

Taken together, our data highlight a convergent dysregulation of excitability-related ion channels pathways in both loss-of-function and mutation-based models, while underscoring mechanistic differences between these conditions. Although the present study is not mechanistic in nature, it establishes a transcriptional framework linking impaired lineage stabilization to altered bioelectrical maturation and identifies downstream pathways that may be useful to prioritize therapeutic targets from future genome-wide analyses.

Premature sodium channel expression may increase excitability before full maturation of calcium handling and potassium stabilization mechanisms, generating asynchronous electrophysiological states. Indeed, such disruptions are linked to neurodevelopmental disorders from epileptic encephalopathy to polymicrogyria with autonomic dysfunction (Zaman et al., 2020), and increased expression has been reported in epilepsy (Guo et al., 2008; Yu et al., 2012). Furthermore, Kv7 channel upregulation may reflect compensatory stabilization response to increased excitability (elevated *SCN3A)*, or dysregulated maturation timing (Huang and Trussell, 2011).

Although the use of a single non-isogenic control iPSC line, not sex-matched with CCHS-derived lines may confound interpretation and represent a limitation of this study, overall, these data suggest disrupted temporal coordination between transcriptional specification and electrophysiological maturation rather than complete lineage failure. Future studies using multiple, isogenic, and sex-matched controls will strengthen the robustness of PHOX2B-related findings.

This phenotype aligns with the clinical features of CCHS, where autonomic neurons are present but functionally impaired (Nobuta et al., 2015; Cui et al., 2024). Dysregulation of PHOX2B-dependent ion channel networks may therefore contribute to altered chemoreflexes or autonomic dysfunction by altering neuronal electrical responsiveness. Indeed, mutations in potassium channels or altered regulation of their function are associated with various disorders, including defective respiratory rhythm and central chemosensitivity (da Silva Junior et al., 2025; Bond et al., 2000; Kessi et al., 2020; Mulkey et al., 2015).

Evidence from KCNQ channel biology supports this possibility. Kv7 channels regulate spontaneous activity of PHOX2B^+^ neurons in the retrotrapezoid nucleus and modulate CO_2_ responsiveness by controlling neuron excitability (Hawryluk et al., 2012; Mulkey et al., 2015). Both LOF and GOF variants in Kv7 channels alter respiratory control and are linked to developmental epileptic encephalopathies, a condition characterized by seizures, developmental delays, breathing problems, and early mortality (Soto-Perez et al., 2023). Developmental studies (particularly from KCNQ2 models) suggest that early M-current reduction can delay neuronal maturation and destabilize network activity indicating a role beyond preventing hyperexcitability (Rosa et al., 2025). Although focused on KCNQ2, similar mechanisms may apply to KCNQ5, expressed in sympathetic ganglia and brainstem nuclei, where altered regulation could shift the balance between tonic and phasic firing and contribute to autonomic dysfunction.

Similarly, RYR2 GOF mutations, producing leaky Ca^2+^ channels and associated to respiratory dysfunction and altered brainstem neurochemistry, may disrupt neurotransmitter signaling and induce spreading depolarization that transiently silences cardiorespiratory circuits, providing a central mechanism for sudden unexpected death in epilepsy (SUDEP) (Aiba et al., 2016).

Because the *in vivo* expression and regulation of *KCNQ5, SCN3A* and *RYR2* in autonomic lineages remain largely unexplored, further studies are needed to define their spatial and temporal expression during ANS development, assess their regulation in *PHOX2B* mutant or KO models, and determine whether PHOX2B-dependent ion-channels regulation is primarily developmental, or adaptive, thereby strengthening the physiological relevance of our findings. Because ion channels represent major pharmacological targets (Bajaj et al., 2020; Abbott, 2020), understanding how PHOX2B regulates their expression may also have therapeutic implications.

### 4.4 SLC44A1 is a downstream metabolic target of the PHOX2B transcriptional program

Although PHOX2B is primarily known as a transcriptional regulator of autonomic neuronal identity, our data indicate that its regulatory program also extends to metabolic pathways supporting neuronal growth.

*SLC44A1* encodes the choline transporter-like protein (CTL-1), a Na^+^-independent intermediate-affinity choline transporter expressed in many cell types, including neurons and oligodendrocytes (Hedtke and Bakovic, 2019). Its major function is choline uptake required for the synthesis of phosphatidylcholine and outer membrane phospholipids which are essential for membrane expansion, organelle biogenesis and mitochondrial integrity. Importantly, genetic disruption of *SLC44A1* causes mitochondrial dysfunction, altered phospholipid homeostasis and progressive neurodegeneration, demonstrating that proper choline transport is critical for neuronal maintenance (Fagerberg et al., 2020).

PHOX2B-dependent regulation of *SLC44A1* therefore suggests a mechanistic link between transcriptional control of neuronal lineage specification and the metabolic requirements of neuronal differentiation. This relationship may be particularly important in neural crest-derived lineages, where rapid membrane expansion and metabolic adaptation accompany neuronal maturation. Interestingly, SLC44A1 has also been detected in extracellular vesicles from neuroblastoma, suggesting relevance for tumor biology (Cheng et al., 2024). The role of SLC44A1 during autonomic neuron development in both control and disease iPSC-derived models is currently under investigation.

## 5 Conclusions

Our work provides new insight into the transcriptional and functional programs controlled by PHOX2B in neuroblastoma cells and expands the range of known PHOX2B targets to include multiple ion channel-encoding genes. By integrating CRISPR-mediated gene knockout, transcriptional profiling, ChIP-seq, and electrophysiological analyses in the IMR32 neuroblastoma cell line, we identify a previously unappreciated role of PHOX2B in modulating neuronal ion flux and excitability. Despite some limitations, due to the use of a single KO clone, and the rescue experiments where PHOX2B re-expression is driven by a heterologous promoter not mimicking the endogenous locus, these findings extend the well-established role of PHOX2B in specifying noradrenergic identity and autonomic circuit assembly to include active control of neuronal excitability modules and metabolic pathways. Moreover, we propose that the KO cell model provides a useful platform to validate new PHOX2B target genes and to investigate pathogenic mechanisms through re-expression of mutant proteins. Identification of downstream genes and pathways dysregulated by PHOX2B dysfunction may uncover therapeutic targets, to be further validated in iPSCs-derived sympathetic neurons, whose modulation could bypass the primary genetic defect and restore altered transcriptional and cellular programs in PHOX2B-associated disorders, including CCHS, NB and HSCR.

## Data Availability

The RNA-sequencing data set generated and analyzed in this study has been deposited in the NCBI database under accession number GEO ID: GSE330047. The ChIP-sequencing datasets are available in the ZENODO repository under DOI: 10.5281/zenodo.20096956.
