# Supplementary material for: CRISPR/Cas9-mediated PHOX2B functional knock-out in IMR32 neuroblastoma cells impairs neuronal excitability through dysregulation of ion channels genes
Source: Front Physiol. 2026 Jun 24;17:1844142. doi: 10.3389/fphys.2026.1844142 (PMC13341513; doi:10.3389/fphys.2026.1844142)
Supplement: Supplementary file 1 [file Table1.docx]

**Supplementary Table 1**

| **Primary antibody** | **dilution** |
| --- | --- |
| Mouse monoclonal anti-PHOX2B (B-11) (Santa Cruz Biotechnology; Cat# sc-376997) | 1:10000 |
| Chicken anti-hPHOX2B (Davids Biotechnologie; Cargnin et al. 2005) | 1:400 |
| Chicken anti-hPHOX2A (Davids Biotechnologie, Benfante et al. 2007) | 1:1000 |
| Mouse monoclonal anti-β-Tubulin (D3U1W) (Cell Signaling Technology; Cat# 86298) | 1:1000 |
| Mouse monoclonal anti-Actin Clone AC-40 (Sigma-Aldrich; Cat# A4700) | 1:1000 |
| Rabbit monoclonal anti-Sox10 (D5V9L) (Cell Signaling Technology; Cat# 89356) | 1:1000 |
| Rabbit monoclonal anti-β3 tubulin (D71G9) XP (Cell Signaling Technology; Cat# 5568) | 1:1000 (WB) 1:100 (IF) |
| Sheep monoclonal anti-Dopamine-β-Hydroxylase (DBH) (N-Terminal) (Sigma-Aldrich; Cat# D-217) | 1:500 |
| Goat Affinity purified polyclonal anti-choline acetyltransferase (ChAT) (Chemicon International; Cat# AB144P) | 1:500 |
| Mouse monoclonal purified IgG Synaptotagmin 1 (Synaptic Systems; Cat# 105 011) | 1:1000 |
| Mouse Ascites Fluid monoclonal anti-syntaxin clone HPC-1 (Sigma-Aldrich; Cat# S 0664) | 1:1000 |
| Rabbit monoclonal anti-Tyrosine Hydroxylase (TH) (E2L6M) (Cell Signaling Technology; Cat# 58844) | 1:50 |
| Mouse monoclonal anti-PHOX2B C-3 (Santa Cruz Biotechnology; Cat# sc-376993) | 1:500 |
|  |  |
| **Secondary antibody** |  |
| Stabilized Goat anti-mouse HRP-conjugated (Pierce; Cat#1858413) | 1:10000 |
| Stabilized Goat anti-rabbit HRP-conjugated (Pierce; Cat#1858415) | 1:10000 |
| Goat anti-chicken IgY HRP-conjugated (Davids Biotechnologie; Cat# 20.0101.05) | 1:100000 |
| Donkey anti-sheep IgG (whole molecule) HRP-conjugated (Sigma-Aldrich; Cat#A 3415) | 1:5000 |
| Rabbit anti-goat IgG (whole molecule) HRP-conjugated (Sigma-Aldrich; Cat# A 5420) | 1:10000 |
| Donkey anti-rabbit IgG Alexa Fluor 488 (Jackson ImmunoResearch; Cat# 711-545-152) | 1:400 |
| Goat anti-mouse IgG DyLight 549 (Jackson ImmunoResearch; Cat# 115-505-146) | 1:200 |

**Table S1. Primary and secondary antibodies used for Western blot and immunofluorescence analyses, along with their corresponding dilutions.**

**Supplementary Table 2**

| **Name** | | | **Sequence** | |
| --- | --- | --- | --- | --- |
| PHOX2B promoter | # FW | 5’- GCT CGG TGC GTA ATG GTG TGG TA - 3 ’ | |  |
|  | # REV | 5’- GGT TGG TCT TAT TGC TGG CGC TT - 3’ | |  |
| # Peak 1 | # FW | 5’- TTC TCT GTG GGG AGA GTG AAT - 3’ | |  |
|  | # REV | 5’- CAG ATG CCA GCA AGA GCA AA - 3’ | |  |
| # Peak 2 | # FW | 5’- CAC ATA GCT AGC TTA CCT CCA GAA - 3’ | |  |
|  | # REV | 5’- ACT AGA AAT GTG ACT CCC CCA AT - 3’ | |  |
| # Peak 3 | # FW | 5’- GCT GAA GTC CTA ATC TCC GGT ATT T - 3’ | |  |
|  | # REV | 5’- GGA CTA GGG CCC ACC AGT AT - 3’ | |  |
| # Peak 4 | # FW | 5’- AGG AAT ATT CAG GCA CAC TCA CT - 3’ | |  |
|  | # REV | 5’- GAC ACC ACC GGC TGT AGA AA - 3’ | |  |
| # Peak 5 | # FW | 5’- TCC ACT GCA GAT GTA ATA AGA ACA A - 3’ | |  |
|  | # REV | 5’- TCA ACT ACA TTG CAT TCA CAA AGG A - 3’ | |  |
| # Peak 6 | # FW | 5’- AGA AGT TGT GGT TGC TTG GC - 3’ | |  |
|  | # REV | 5’- GGA TTT CAC TTT TTA CTG CAT GGT - 3’ | |  |
| # Peak 7 | # FW | 5’- AGG CAA TTG TCC TGA GCA AAT TAA A - 3’ | |  |
|  | # REV | 5’- GTT CCC ATG GAA TCT GTT TCT TCA - 3’ | |  |
| # Peak 8 | # FW | 5’- TGC TGG AGC ATT TAA ATC CGT TG - 3’ | |  |
|  | # REV | 5’- TCC TTT ACC AGA AAC CTC AAA AAC A - 3’ | |  |
| # Peak 9 | # FW | 5’- CCC ATT GCA CCT TCA ACA CAA TA - 3’ | |  |
|  | # REV | 5’- CAG TAC AGT GAG AGA ATT GCC CA - 3’ | |  |
| # Peak 10 | # FW | 5’- TGA TGA AAT GAG ATG ATG CCA GAT - 3’ | |  |
|  | # REV | 5’- TTA GTT ACT AGG GAC ACA GTG CT-3’ | |  |
| # Peak 11 | # FW | 5’- GCT GTG ATG GAA TAT GGA GCC TA - 3’ | |  |
|  | # REV | 5’- TCA ATC TTT CCC CAT TGT GAT CCT - 3’ | |  |
| # Peak 12 | # FW | 5’- CCT TGG ACA CTT GCT TAC CCT AT - 3’ | |  |
|  | # REV | 5’- TTC CCA CCT AAA TTT GCC AAT GC - 3’ | |  |
| # Peak 13 | # FW | 5’- GGT GCT CTG AAG TCC GTA ACT AA - 3’ | |  |
|  | # REV | 5’- TGT TCA GTA AAC CAC ACC TAA ACT - 3’ | |  |
| # Peak 14 | # FW | 5’- GGC AGG TTA TAC CAT TGT AGG CT - 3’ | |  |
|  | # REV | 5’- TCC TGT CTT CTG TTC GAT CAC TG - 3’ | |  |
| Negative CTRL region (*PHOX2B* 3’- UTR) | # FW | 5’- GTA GGC CCA AGG CTA TTG TCG TCG CT - 3’ | |  |
|  | # REV | 5’- GTA GGA GTG GGG TTG AAA TGA GGG CG - 3’ | |  |

**Table S2. Sequences of the primers used in the conventional ChIP followed by SYBR-Green based qRT-PCR.** All the primers have been used at a 300 nM final concentration.
