# Supplementary material for: CRISPR/Cas9-mediated PHOX2B functional knock-out in IMR32 neuroblastoma cells impairs neuronal excitability through dysregulation of ion channels genes
Source: Front Physiol. 2026 Jun 24;17:1844142. doi: 10.3389/fphys.2026.1844142 (PMC13341513; doi:10.3389/fphys.2026.1844142)
Supplement: Supplementary file 2 [file DataSheet1.docx]

**Supplementary Figure 1**

**
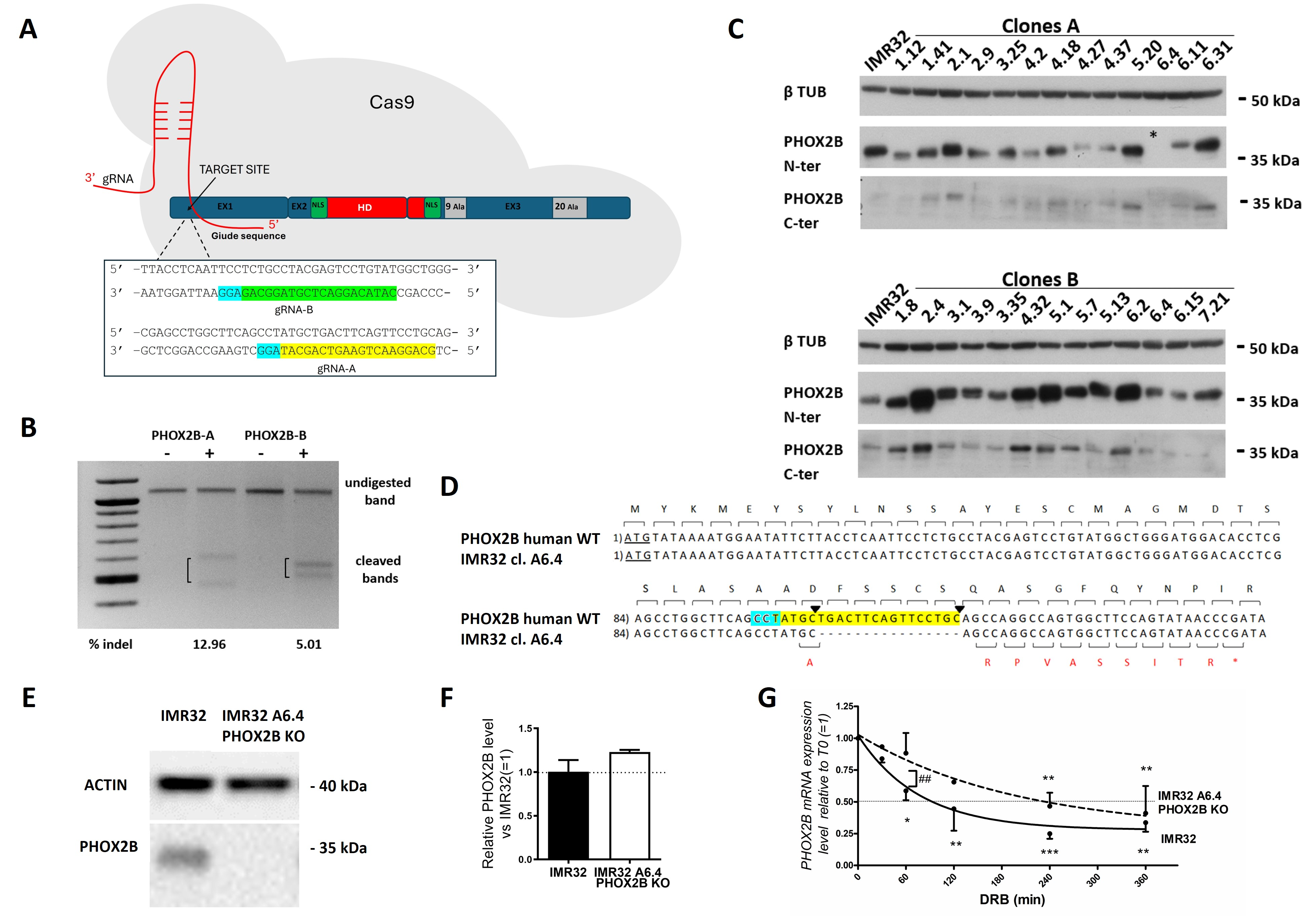
**

**Figure S1. Generation of the *PHOX2B* knock out cell line. A:** *top*: Schematic representation of the *PHOX2B* gene and the CRISPR/Cas9 machinery. The domain structure of full-length PHOX2B is shown, including the homeodomain (HD, red), the NSL domains (green), and the two polyalanine stretches (9 Ala and 20 Ala in grey). The gRNA (in red) target site is located at the beginning of exon1 (EX1). *Bottom*: close-up view of the two-gRNAs target sequences. Immediately 3’ to the target sequence (*PHOX2B-A* in yellow and *PHOX2B-B* in green respectively) is the trinucleotide spacer adjacent motif (PAM in cyan). **B:** Gel image of the cleavage detection assay (Life Technologies). After transfection with CRISPR All-in-one vectors (*PHOX2B-A*, and -*B*), PCR amplification and re-annealing, samples were treated with (+) or without (-) Detection Enzyme and run on a 2% agarose gel. Nuclease treatment produced specific cleaved bands (brackets). The cleavage efficiency, expressed as percentage of indel, was calculated for each sample as a difference between undigested band and the sum of the cleaved bans. **C:** A representative Western blot analysis of clones generated by the transfection of All-in-one vectors containing the gRNA *PHOX2B-A* and -*B*. Each numbered lane corresponds to an individual clone. The asterisk indicates clone A6.4 that does not express PHOX2B protein. Protein extracts of the IMR32 cell line was used as positive control. Antibodies targeting the N- And C-terminal regions were used to confirm loss of expression. **D**: Sequence alignment of a portion of PHOX2B exon 1, comparing the WT human sequence (human *PHOX2B* WT), and the edited IMR32 cell lines cl. A6.4. The putative ATG start codon is underlined, and the 3-nt PAM sequence and the 20-nt target sequence are highlighted in cyan and yellow, respectively. Clone A6.4 carries a 16-nt deletion at the cleavage site (arrows). The WT amino acid sequence is displayed above the nucleotide alignment, while amino acid changes in clone A6.4 are shown below and highlighted in red. A premature stop codon is indicated by an asterisk. **E**: Representative western blot of total cell lysates from native IMR32 and the IMR32 A6.4 *PHOX2B* KO clone. 20 μl of protein extract were separated by 10% SDS-PAGE and transferred onto a nitrocellulose membrane. Actin was used as a loading control. **F**: qPCR analysis of *PHOX2B* mRNA expression in native IMR32 (black bar) and IMR32 A6.4 *PHOX2B* KO clone (white bar). Expression values were normalized to *GAPDH* . The bars are the mean values ± SD (error bars) of at least three independent experiments, performed in triplicate. Results are expressed as fold change relative to IMR32 native cells (=1) using the 2^−ΔΔCT^ method. **G**: *PHOX2B* mRNA half-life in IMR32 and IMR32 A6.4 *PHOX2B* KO cells following transcriptional inhibition with 75 μM DRB. RNA was isolated at the indicated time points, and relative *PHOX2B* mRNA levels were measured by RT-qPCR. Data are represented as mean values ± SD (error bars) of three independent experiments performed in triplicate and normalized to the endogenous *GAPDH* gene. *PHOX2B* mRNA levels at each time point were compared to those in native cells (initial level, T0 = 1) and are expressed as fold induction calculated using the 2−^ΔΔCT^ method. Non-linear regression analysis was used to calculate one-phase exponential *PHOX2B* mRNA decay. ***p < 0.001, **p < 0.01 and *p < 0.05, statistically significant differences in *PHOX2B* mRNA levels between each time point and T0 (one-way ANOVA, Tukey's test). ##p < 0.01 statistically significant differences in *PHOX2B* mRNA levels between IMR32 A6.4 PHOX2B KO cells and native cells after 30 minutes of DRB treatment (Student’s t-test).
