# Supplementary material for: CRISPR/Cas9-mediated PHOX2B functional knock-out in IMR32 neuroblastoma cells impairs neuronal excitability through dysregulation of ion channels genes
Source: Front Physiol. 2026 Jun 24;17:1844142. doi: 10.3389/fphys.2026.1844142 (PMC13341513; doi:10.3389/fphys.2026.1844142)
Supplement: Supplementary file 3 [file DataSheet2.docx]

**Supplementary Figure 2**


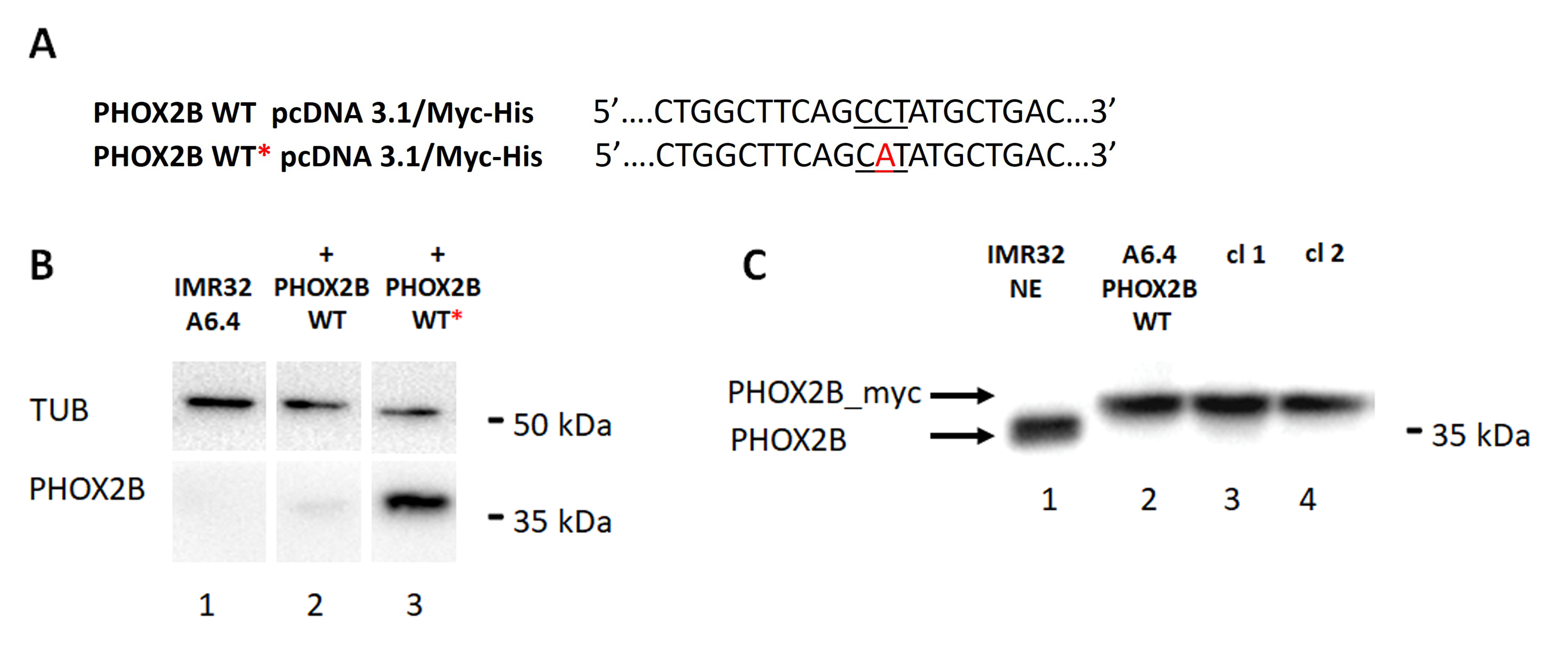


**Fig. S2 generation of stable A6.4 clones re-expressing PHOX2B protein**. **A**: schematic representation of the *PHOX2B* sequence at the PAM site (underlined) in the expression vector pcDNA3.1/Myc-His, shown before (top) and after (bottom) the single-nucleotide substitution introduced to prevent CRISPR targeting. The modified nucleotide is highlighted in red. **B:** Representative Western blot showing PHOX2B protein levels in IMR32 A6.4 *PHOX2B* KO cells (lane 1), and after transient transfection with PHOX2B WT pcDNA3.1/Myc-His (lane 2) or mutagenized PHOX2B WT* pcDNA3.1/Myc‑His (lane 3). Only the mutagenized construct restores PHOX2B expression in A6.4 *PHOX2B* KO cells. Tubulin (TUB) serves as loading control. **C:** Representative Western blot of stable clones (cl 1 and cl2, lanes 3 and 4) generated by transfection with the mutagenized PHOX2B WT* pcDNA3.1/Myc-His construct in IMR32A 6.4 cells. IMR32 nuclear extract (NE, lane 1) and transient transfection of PHOX2B WT* pcDNA3.1/Myc-His plasmid in IMR32 A6.4 cells (lane 2) are shown as controls. Bands corresponding to endogenous PHOX2B and PHOX2B‑myc are indicated.
