# Supplementary material for: CRISPR/Cas9-mediated PHOX2B functional knock-out in IMR32 neuroblastoma cells impairs neuronal excitability through dysregulation of ion channels genes
Source: Front Physiol. 2026 Jun 24;17:1844142. doi: 10.3389/fphys.2026.1844142 (PMC13341513; doi:10.3389/fphys.2026.1844142)
Supplement: Supplementary file 4 [file DataSheet3.docx]

**Supplementary Figure 3**

**Figure S3.** **qPCR analysis of PHOX2A mRNA level.** Re-expression of PHOX2B partially rescue the over-expression of PHOX2A. ** and ****p< 0.01 and 0.0001 respectively versus KO cells (one-tailed Student’s *t-test*).
