# Supplementary material for: CRISPR/Cas9-mediated PHOX2B functional knock-out in IMR32 neuroblastoma cells impairs neuronal excitability through dysregulation of ion channels genes
Source: Front Physiol. 2026 Jun 24;17:1844142. doi: 10.3389/fphys.2026.1844142 (PMC13341513; doi:10.3389/fphys.2026.1844142)
Supplement: Supplementary file 5 [file DataSheet4.docx]

**Supplementary Figure 3**


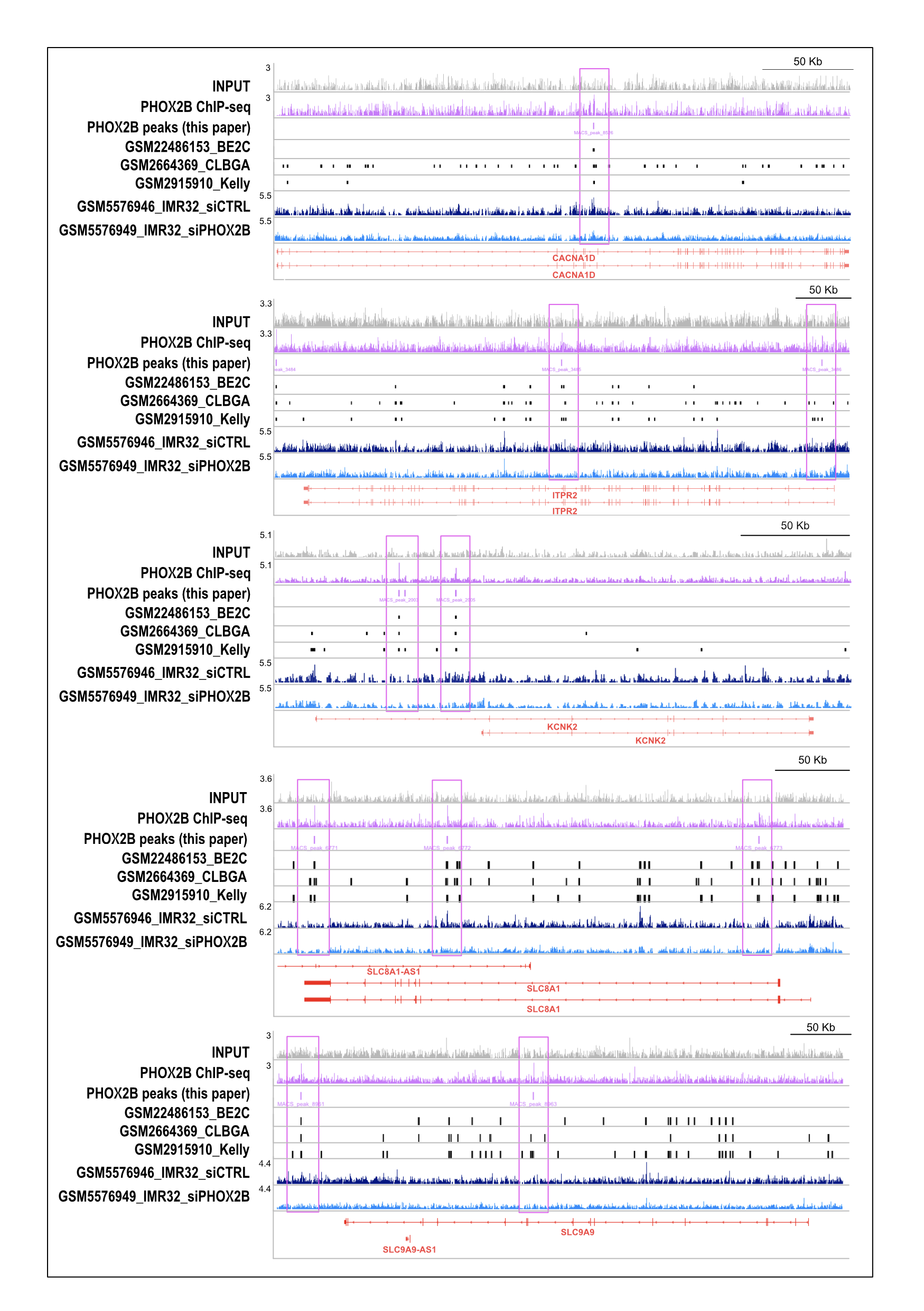


**Fig. S3 Genome browser views of PHOX2B binding at** *CACNA1D*, *ITPR2*, *KCNK2*, *SLC8A1*, and *SLC9A9* genes, compared with published ChIP-seq datasets in other neuroblastoma cell lines (Boeva et al (2017, Durbin et al. 2018). Peaks shared between our dataset and published datasets are boxed in magenta, indicating concordant PHOX2B binding sites.
