# Supplementary material for: CRISPR/Cas9-mediated PHOX2B functional knock-out in IMR32 neuroblastoma cells impairs neuronal excitability through dysregulation of ion channels genes
Source: Front Physiol. 2026 Jun 24;17:1844142. doi: 10.3389/fphys.2026.1844142 (PMC13341513; doi:10.3389/fphys.2026.1844142)
Supplement: Supplementary file 6 [file DataSheet5.docx]

**Supplementary Figure 5**


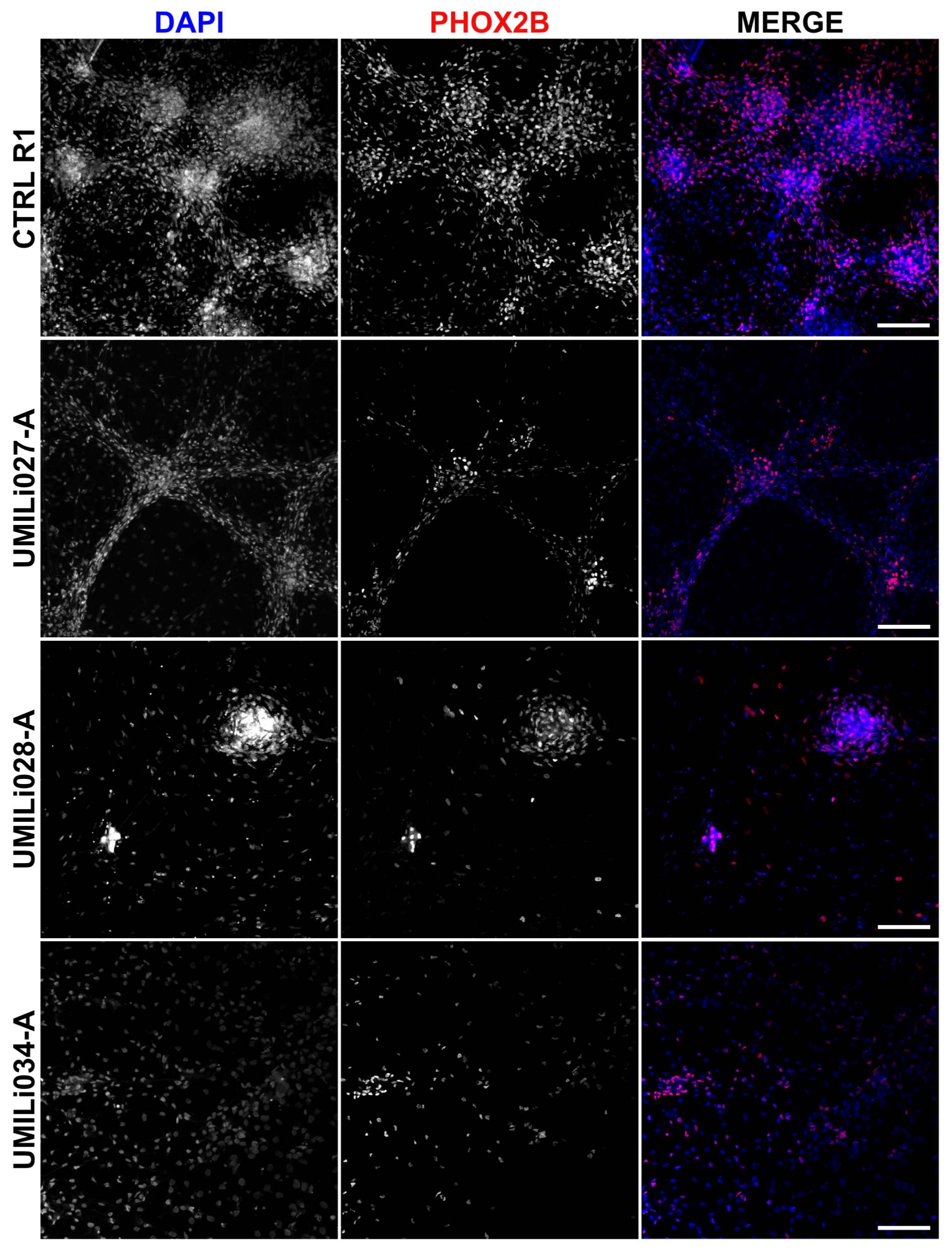


**Figure S5.** DAPI counterstaining confirm the predominantly nuclear localization of PHOX2B in CTRL R1 as well as in mutant lines. Scale bar = 100 μm.
